# RNA-seq: technical variability and sampling

**DOI:** 10.1186/1471-2164-12-293

**Published:** 2011-06-06

**Authors:** Lauren M McIntyre, Kenneth K Lopiano, Alison M Morse, Victor Amin, Ann L Oberg, Linda J Young, Sergey V Nuzhdin

**Affiliations:** 1 Department of Molecular Genetics and Microbiology, University of Florida, Gainesville, Florida, USA; 2 Department of Statistics, University of Florida, Gainesville, Florida, USA; 3 Department of Health Sciences Research, Division of Biomedical Statistics and Informatics, Mayo Clinic, Rochester, Minnesota, USA; 4 Molecular and Computational Biology, University of Southern California, California, USA

## Abstract

**Background:**

RNA-seq is revolutionizing the way we study transcriptomes. mRNA can be surveyed without prior knowledge of gene transcripts. Alternative splicing of transcript isoforms and the identification of previously unknown exons are being reported. Initial reports of differences in exon usage, and splicing between samples as well as quantitative differences among samples are beginning to surface. Biological variation has been reported to be larger than technical variation. In addition, technical variation has been reported to be in line with expectations due to random sampling. However, strategies for dealing with technical variation will differ depending on the magnitude. The size of technical variance, and the role of sampling are examined in this manuscript.

**Results:**

In this study three independent Solexa/Illumina experiments containing technical replicates are analyzed. When coverage is low, large disagreements between technical replicates are apparent. Exon detection between technical replicates is highly variable when the coverage is less than 5 reads per nucleotide and estimates of gene expression are more likely to disagree when coverage is low. Although large disagreements in the estimates of expression are observed at all levels of coverage.

**Conclusions:**

Technical variability is too high to ignore. Technical variability results in inconsistent detection of exons at low levels of coverage. Further, the estimate of the relative abundance of a transcript can substantially disagree, even when coverage levels are high. This may be due to the low sampling fraction and if so, it will persist as an issue needing to be addressed in experimental design even as the next wave of technology produces larger numbers of reads. We provide practical recommendations for dealing with the technical variability, without dramatic cost increases.

## Background

RNA-seq (high throughput sequencing of the transcriptome) has the potential to transform the way we study gene structure and expression [1]. The ability to identify novel exons and splice sites [2-6] is just the beginning. Although there have been claims that RNA-seq is more sensitive [7] and has a larger dynamic range [3] than a microarray, these claims are now being challenged [8]. For all the optimism surrounding RNA-seq, there is growing evidence that estimating gene expression in an RNA-seq environment is not straightforward. In addition, technical variance, sequencing bias and mapping bias have been reported [9-11].

The power and promise of RNA-seq technology demand our attention. New papers on experimental design [12] will lead the way to more thoughtful experimentation. One important component in careful study planning is an understanding of the different sources of variability so they can be accounted for in the experimental design [13]. Variability can occur at many levels, both biological and technical. Biological variability is unaffected by the technology, as genotypes, individuals and even cell types vary regardless of how they are measured. Bullard et al. (2010) [14] examined the effects of library construction, flow cell and lane on detection of differential expression. The data used there was commercial grade RNA and the samples compared expected to be very divergent. They concluded that the biological variation was larger than technical variation, underscoring the importance of including biological replicates in the study design.

The observation that biological variability is large does not imply that technical variability should be ignored in the experimental design of a study. High levels of noise in a technology must be considered during experimental design [13]. Marioni et al. (2008) [15] demonstrate clearly that technical variability is not different from what is expected due to random noise (sampling error). Yet, the magnitude of the sampling error is not explicitly discussed. One particular feature of deep sequencing is that even though there are millions of reads, the proportion of the mRNA that is actually sequenced is low. Given a Solexa/Illumina RNA-seq library of 10 nM concentration with a mean insert size of 250 bp and a volume of 400 uL, the number of molecules in a typical RNA library is estimated to be 2.408 × 10^12^. On the Solexa/Illumina GAIIx, approximately 30 million of the total possible molecules are sampled in a given lane. This represents approximately 0.0013% of the total number of available molecules. Even with the anticipated increase in the next generation of technology, the sampling fraction is will be less than 0.004%. The impact of such a low sampling fraction on the statistical properties of RNA-seq is the focus of this manuscript. We address the following questions: *1) Does a substantial amount of technical variability exist? 2) Is the impact of technical variability the same for all levels of coverage?*

The study presented here considers technical variation in three experiments using Solexa/Illumina technology. Although the number of exons consistently detected is improved by increasing the number of mappable reads, the detection of exons with low levels of coverage is inconsistent among technical replicates. Inconsistent detection of exons is most pronounced for exons with average coverage of less than 5 reads per nucleotide. In addition, there can be substantial disagreement in the estimated level of expression among technical replicates, even when the coverage is high. Although the technical variability is not unexpected, technical variability cannot be ignored in RNA-seq experiments.

## Methods

### Experiments

**Figure 1:**
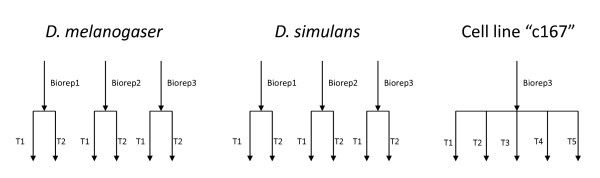
**Experimental Design**. A figure showing the design of the three experiments evaluated here. Biological replicates are separate individuals used for library construction. Technical replicates for the *D. melanogaster *female heads and *D. simulans *male heads data are a single library run on multiple lanes. For *D. melanogaster *c167 cell lines the exact nature of the technical replication is uncertain.

Experiment 1: Three independent samples of *D. melanogaster *female heads were collected with each sample representing a unique pool of biological material. Each sample was prepared according to manufacturer's instructions and then the same library was run on two lanes of a Solexa/Illumina flow cell, resulting in two technical replicates for each biological replicate, runs were 36 base-pair paired end. Experiment 2: Three independent samples of D. *simulans *male heads were collected with each sample representing a unique pool of biological material. Each sample was prepared according to manufacturer's instructions and then the same library was run on two lanes of a Solexa/Illumina flow cell, resulting in two technical replicates for each biological replicate, runs were 36 base-pair paired end. (Data will be deposited to the SRA) Experiment 3: One sample of *D. melanogaster *cell line c167 was run on 5 lanes yielding 5 technical replicates for 1 biological replicate (from modENCODE experiment GSE17107) [16]. Data from these experiments were 36 bp reads. The experimental design for experiments 1 - 3 are outlined in Figure 1. All samples were run using Solexa/Illumina paired end procedures. The relationship of lanes (same or independent flow cells) is unknown for all three experiments. The first two experiments were conducted by Joe Dunham and Michele Arbeitman at USC and sequenced at Oregon State core facility while the third experiment was conducted by Susan Celniker at Lawrence Livermore national laboratory (SRA009944).

### Simulation

**Figure 2:**
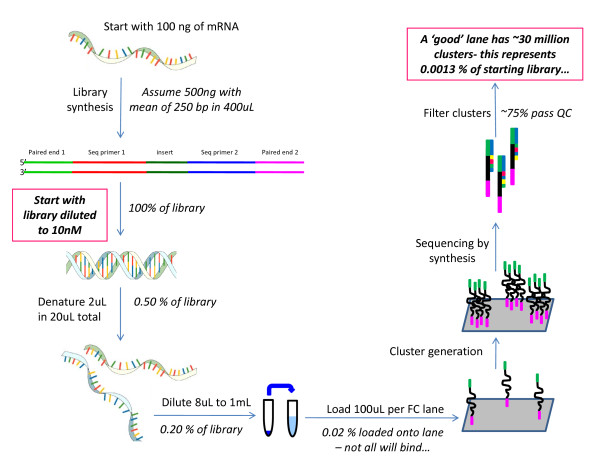
**Library construction and sequencing**. Beginning with 100 ng of mRNA the manufacturer's protocol is used to estimate a sampling fraction.

The number of molecules present in the library is estimated to be 2.408 × 10^12^. This estimate was derived using the Illumina protocol. Specifically, the starting material was assumed to be 100 ng of mRNA that then resulted in 500 ng of library with an insert size of 250bp in a volume of 400 uL for a final library concentration of 10 nM. The number of pmoles is therefore 4 [(10 nmol/1L) (1L/1000 mL)(1 mL/1000 uL)(400 uL)(1000 pmol/1 nmol)]. The number of molecules is found by multiplying Avogadro's number which is molecules/moles and adjusting for units [4 pmole)/1000/1000/1000/1000*6.02E + 023] to give 2.408 × 10^12 ^molecules. On the current Solexa/Illumina technology, the GAIIx, approximately 30 million of the total possible molecules are sampled in a given lane. This represents approximately 0.0013% (30,000,000/2.408 × 10^12^) of the total number of available molecules for analysis (Figure 2). To understand how random sampling may produce variation in technical replicates in an RNA-seq experiment, a small simulation of two technical replicates was conducted. We assume that the approximately 2.408 × 10^12 ^molecules of mRNA were transcribed from 20,000 genes. Gene expression for the 20,000 genes was modeled using a gamma density function with shape parameter 2 and scale parameter 1. This ensures that many genes will have a small number of molecules assigned to them and some genes will have a large number of molecules assigned to them. The range of the gamma density function was examined over the interval from 0 to 17. The integers from 0 to 17 represent bins of genes with similar relative levels of expression. To simulate relative expression for each of the *i *= 1, 2, ..., 20,000 individual genes, a random integer (*S*_*i*_) from a discrete uniform distribution between 0 and 17 were drawn and the value of the gamma density *t*_*i *_was calculated at *S*_*i*_. The number of total molecules of mRNA assigned to gene *i*, *g*_*i*_, was, N_i_= *t*_*i*_/Σ(*t*_*i*_) * 2.408 × 10^12 ^rounded to the nearest integer. Σ(g_i_) = Z. Due to the rounding, Z was approximately, but not exactly equal to2.408 × 10^12^. For each g_i _with N_i _molecules a unique integer between 1 and Z was assigned, resulting in a unique association between an integer and a gene. To simulate the sampling of molecules of mRNA for each technical replicate, 30 million random integers between 1 and *Z *were sampled.

### Exon definitions

For this paper exons, rather than transcripts, were used. Estimating levels for individual transcript isoforms in RNA-seq data can be quite complex as the observed data can be a mixture of isoforms [17,18]. Transcript assembly and abundance estimation from RNA-Seq reveals thousands of new transcripts and switching among isoforms. To focus on the issue of technical replication, isoform estimation was not undertaken. Exons as defined by Flybase (http://www.flybase.org) version 5.4 [19] were mapped onto the genome. Overlapping exons in the same genome region were combined. A single result per genomic region was recorded for each exon in that region (Additional File 1, Additional File 2). For example, for exons with alternative start/end sites the longest genomic region was used to represent an exon. The mapping between exons and the genome region was done via a perl script (Additional File 3). 54,607 of the 60,277 genomic position exons represent uncomplicated annotation regions. However, to ensure that results are not dependent on the exon definition, the observed data were used to derive exons. Consensus contigs were defined as contiguous regions with a per nucleotide read depth greater than or equal to 3 for all lanes within a particular experiment (Additional File 4, 5). This definition results in the evaluation of genomic regions for which RNA-seq data are available for all replicates of a particular experiment. Consensus contigs and their positions for these data are listed in Additional Files 6, 7, 8.

### Mapping reads

Reads spanning junctions pose a problem. Each lane of data was first evaluated with TopHat [20] to create a set of read-supported junctions. Bowtie [21] was used as the main tool for read alignment. Alignments were run with the "best" option to ensure that reported singleton alignments have the optimal score. The "tryhard" flag was also used to induce Bowtie to spend more time backtracking for hard-to-align reads. Reads aligning more than 10 times were removed from consideration. First, a paired-end alignment was run. Unaligned reads from this step were aligned unpaired. Remaining unaligned reads were mapped to the junctions from TopHat. For *D. simulans *samples, reads were also aligned to *D. simulans *genomic reference sequence [22]. Bowtie is fast but cannot handle in/dels or reads that align partially to the reference. Reads remaining unaligned at this point were aligned with LAST [9]. Results were combined into a single alignment file in SAM format and reported versus single reference (the Flybase v5 D.mel reference [19]). Analysis of read coverage was done with SAMtools [23]. For each lane the resulting pileup file was the basis for all further calculations and the remaining unmapped reads were discarded.

### Detection and Quantitation

Each exon was projected onto each pileup file. If at least one read maps to a particular exon then that exon was said to be detected. RPKM which is the (number of reads in a window/length of the window)*C/mappable reads), where C is an estimate of the maximum number of reads [3] was used to estimate the quantity of each exon.

### Analysis

To understand variability among replicates it is necessary to understand how well replicates agree, not how well they correlate [24-26]. Agreement between categorical variables can be calculated as the proportion of observations that share the same category divided by the total number of observations [25]. If the number of observations in each category is unequal then two measurements may agree by chance more frequently than estimated using a simple agreement. Kappa calculates the agreement between two replicates and adjusts for chance agreement [25]. The Kappa statistic gives a measure of agreement and the value 1 represents perfect agreement [25]. The most basic question is whether exons can be consistently detected between replicates. The agreement of technical and biological replicates on detection of exons was calculated using both simple agreement and Kappa statistics.

For ordinal scales, a weighted Kappa allows for distance in categories to be used in the assessment of agreement and is interpreted in the same way as the Kappa [25]. The RPKM was grouped into 3 levels undetected/low-medium/high where high levels were defined as RPKM > 20. Alternatively RPKM was grouped into 9 levels using a log scale as follows: Zero reads, RPKM less than 10, 20, 40, 80, 160, 320, 1000 and greater than 1000 were the ordinal categories used. Simple agreement (two replicates having the same level of coverage) and weighted kappa statistics were calculated. Examination of the frequency table between the technical replicates can give insight into whether the disagreements are solely a function of the level of digital gene expression.

Correlation coefficients have often been used to describe agreement. However, correlation does not reflect agreement [24-26]. Spearman's correlation coefficient quantifies how well the relationship between two variables, (Y,X), can be described using a monotonic function. If one estimate is exactly twice the size of the other estimate then the correlation will be 1. However, these two measures do not agree. In addition to the scale factor, correlations depend upon the range of values observed. A large range of values will produce a higher correlation than a narrow range of values. In comparisons of whole genome expression the range is extremely large. A further problem is the large number of data points makes the linear trend unlikely to be influenced by strong disagreement for some exons.

Bland-Altman [24,26] proposed a method for visualizing data and providing insights into agreement. These plots are known as MVA or MA (minus versus average) plots in the gene expression literature and have been utilized intensively to assess presence of abundance-dependent bias [27]. The plot is defined as follows: on the X axis is the average of the two measures (in this case the technical replicates) and on the Y axis is the difference between the two measures. These plots were constructed for exons for all combinations of technical replicates where there are non-zero measurements in both replicates. An absolute difference of 10 has a different implication for agreement if the RPKM is 20 than if the RPKM is 300. For that reason, Bland-Altman plots were also made on the natural log scale. That is, the natural log of the RPKM was calculated for each technical replicate where the scale is considered to be additive rather than multiplicative and then used for computing the average and the difference.

Coverage plots [28] were constructed to examine whether exon length was associated with missing values. To formally test whether exon abundance and/or length is related to whether an exon is present in all or only a portion of the technical replicates, an analysis of the dataset for the cell line c 167 was conducted. Only exons that were observed in at least one technical replicate were considered. The dichotomous response of "in all technical replicates" versus "not in all technical replicates" was modeled using logistic regression. Formally, Let Y_i _= 1 if the i^th ^exon is not present in all technical replicates, 0 otherwise. The model

was fit, where a_i _is the abundance of the i^th ^exon and l_i _is the length of the i^th ^exon and i = 1,..,n where n is the number of exons observed in at least one technical replicate. The average RPKM, taken across all technical replicates was used as the measure of abundance for that exon and the length of the exon in base pairs was used as the measure of exon length.

## Results

For the three experiments, the number of mappable reads per lane varies among the technical replicates and the number of exons detected increases with the number of mappable reads (Table 1). The exons that are detected vary between technical and biological replicates (Table 1). Many exons are observed/present in one technical replicate but unobserved/absent in the other technical replicate. There was no exception to this observation in any comparison made (Table 2). The agreement (Kappa) in detection between technical replicates ranges from 0.63-0.81. Lanes with higher coverage have better agreement in detection, but even with the higher coverage rates, the number of exons detected varies. The comparison with the least discrepancy had ~3,600 exons missing in one of the two technical replicates and several had more than 5,000 missing (Table 2). Among biological replicates discrepancies were larger than for technical replicates in that same experiment (Table 2). The simulation study shows that this variability in detection can be explained due to random noise from a very small sampling fraction (0.0013%). A second simulation (not shown here) based upon the multinomial model came to the same conclusion. In the simulation, and in the real data examined here, discrepancies in detection are largely due to exons with coverage per nucleotide of less than 5 in the technical replicate in which they are detected (Table 2, Additional File 9). This indicates that higher coverage increases the consistency of detection across technical replicates. Unfortunately, the vast majority of exons are observed at low coverage (Table 1).

**Table 1:** Mappable reads per lane in each of the three experiments.

Experiment	BR	TR	Mappable Reads	Exons detected	Exons with an average coverage of more than 5 reads per nucleotide	Contigs present in all samples of each experiment
c167	1	1	5888686	39156	13432	19248

c167	1	2	5951769	39202	13517	19248

c167	1	3	7146461	39954	15684	19248

c167	1	4	7544117	40201	16355	19248

c167	1	5	7377032	40120	16089	19248

D. sim.	1	1	5174398	45878	14517	20339

D. sim.	1	2	4979485	45808	13912	20339

D. sim.	2	1	27595266	51701	35303	20339

D. sim.	2	2	28691914	51857	35942	20339

D. sim.	3	1	27601233	51834	34968	20339

D. sim.	3	2	27748704	51822	35008	20339

D. mel.	2	1	10584341	48114	13396	17864

D. mel.	2	2	13399722	49073	19916	17864

D. mel.	3	1	12065885	48281	14794	17864

D. mel.	3	2	11794255	48319	17961	17864

D. mel.	4	1	10375138	47812	15718	17864

D. mel.	4	2	9283979	47460	14344	17864

BR indicates a biological replicate and TR indicates a technical replicate. The experiments are described in Figure 1. There are a total number of 60,277 exons corresponding to distinct genomic regions in Flybase 5. In this replicate 14,972 replicates disagree by at least 1 log and 204 disagree by 2 or more logs.

**Table 2:** Agreement between technical replicates and biological replicates for RPKM measured on FB 5.4 exons (n = 60,277).

Experiment	Comparison	Number of exons in common	Exons detected in only one of the two replicates	Kappa for detection	Detected in one replicate, RPKM > 20 in the other replicate	Kappa on a 3 level scale	Kappa on a 9 level log scale	Number of exons where disagreement is greater than 2 logs	Number of exons that disagree 1 log or more
c167	TR1-TR2	36602	5154	0.812	0	0.854	0.886	128	11596

c167	TR1-TR3	36937	5236	0.808	0	0.853	0.888	119	11395

c167	TR1-TR4	37102	5153	0.810	0	0.854	0.887	111	11562

c167	TR1-TR5	37037	5202	0.808	0	0.853	0.885	112	11686

c167	TR2-TR3	36974	5208	0.808	0	0.855	0.889	104	11285

c167	TR2-TR4	37102	5199	0.808	1	0.853	0.886	95	11600

c167	TR2-TR5	37039	5244	0.807	2	0.851	0.884	102	11779

c167	TR3-TR4	37514	5127	0.809	0	0.857	0.892	76	11026

c167	TR3-TR5	37470	5134	0.809	0	0.856	0.891	98	11051

c167	TR4-TR5	37626	5069	0.811	0	0.858	0.893	58	10869

D. mel..	BR2:TR1-TR2	46123	4941	0.738	67	0.779	0.801	204	14972

D. mel..	BR3:TR1-TR2	45942	4716	0.754	46	0.783	0.798	297	15122

D. mel..	BR4:TR1-TR2	45310	4652	0.767	110	0.814	0.848	105	12206

D. sim.	BR1:TR1-TR2	43614	4458	0.797	343	0.834	0.861	317	14590

D. sim.	BR2:TR1-TR2	49941	3676	0.748	0	0.864	0.909	2	8530

D. sim.	BR3:TR1-TR2	49983	3690	0.746	2	0.861	0.905	6	8648

D. mel..	BR2-BR3	45675	5045	0.739	62	0.803	0.843	58	11266

D. mel..	BR2-BR3	45715	5003	0.741	70	0.776	0.796	289	15304

D. mel..	BR2-BR3	46274	4806	0.744	41	0.779	0.797	271	15149

D. mel..	BR2-BR3	46381	4630	0.753	50	0.815	0.852	70	11775

D. mel..	BR3-BR4	45612	4967	0.748	84	0.774	0.787	444	15891

D. mel..	BR3-BR4	45387	4869	0.750	96	0.773	0.785	446	15938

D. mel..	BR3-BR4	45723	4685	0.759	88	0.803	0.831	176	13434

'D. mel..	BR3-BR4	45450	4879	0.752	108	0.797	0.828	201	13718

D. mel..	BR2-BR4	45459	5008	0.744	113	0.774	0.789	405	15754

D. mel..	BR2-BR4	45200	5174	0.739	108	0.771	0.790	401	15808

D. mel..	BR2-BR4	46067	4751	0.750	75	0.801	0.834	154	13104

D. mel..	BR2-BR4	45846	4841	0.749	89	0.799	0.832	152	13312

D. sim.	BR1-BR2	45442	6695	0.645	362	0.640	0.654	4936	29729

D. sim.	BR1-BR2	45440	6855	0.635	346	0.640	0.658	4687	29659

D. sim.	BR1-BR2	45341	6827	0.639	395	0.639	0.654	4909	29709

D. sim.	BR1-BR2	45375	6915	0.633	402	0.640	0.658	4665	29629

D. sim.	BR1-BR3	45444	6824	0.637	326	0.640	0.655	4835	29452

D. sim.	BR1-BR3	45464	6772	0.640	346	0.642	0.655	4814	29486

D. sim.	BR1-BR3	45368	6906	0.634	355	0.640	0.656	4761	29404

D. sim.	BR1-BR3	45368	6894	0.635	343	0.640	0.656	4704	29438

D. sim.	BR2-BR3	49844	3847	0.737	1	0.828	0.865	104	12328

D. sim.	BR2-BR3	49835	3853	0.737	0	0.830	0.865	98	12327

D. sim.	BR2-BR3	49955	3781	0.739	2	0.829	0.865	113	12376

D. sim.	BR2-BR3	49891	3897	0.731	0	0.828	0.865	95	12413

All technical replicates were compared between biological replicates. For example, BR1 TR1 was compared to BR2 TR1, BR2 TR2, and BR2 TR3 for all possible pairwise comparisons. Agreement in whether exons are detected (defined as at least one read mapping to the exon), detected at a low level (0 < RPKM < 20), and detected at a high level (RPKM > 20) and agreement on a 9 level ordinal scale as follows: RPKM less than 10, 20, 40, 80, 160, 320, 1000 and greater than 1000. Agreement was measured using a kappa coefficient. The number of exons where disagreement is greater than 2 logs and greater than one log are also given. Agreement for common contigs is in Additional file 9, Supplementary Table S3

Additionally, the length of the exon may contribute to the discrepancy. To examine this, coverage plots were constructed (Additional File 4). Coverage plots have a solid line if the exon was detected in the corresponding biological and technical replicate and a blank space if the exon was not present. The plots were conducted by sorting the exons by length. Subsequent plots were made for exons surrounding a given percentile of exon length. For exons that are detected inconsistently among replicates there seems to be no trend associated with the length. Plots of the 10th percentile appear to be similar to the 20^th^, 50^th ^and 90^th ^percentile (Additional Files 10). The coverage plots do not take mRNA abundance into account. To test whether exon length and abundance, and the interaction between them contribute to differences in detection among technical replicates a logistic regression model was used. The interaction between length and abundance was significant (estimate, -0.0136; p < 0.001). This means that the relationship between length and abundance differs between highly abundant and infrequently observed exons. For infrequently observed exons, longer exons are more likely to be consistently detected than shorter exons. This pattern is not observed in highly abundant exons. It must be noted that this is a trend, and that some long exons are missing among some technical replicates and some short exons are present in all. Although main effects are often difficult to describe in the presence of interactions, in this case, it is clear that regardless of the exon length lower levels of abundance are less likely to be consistently detected (estimate -0.25; p < 0.001).

**Figure 3:**
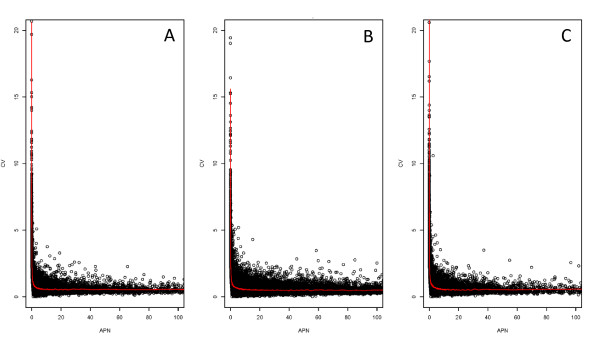
**Coefficient of variation (CV) plotted on Y axis and average depth per nucleotide (APN) on X axis**. Points with average depth of greater than 1000 are not displayed. Panel A is *D. simulans *BR2 TR2. Panel B is *D. melanogaster *female heads BR2 TR1. Panel C is TR1 for cell line c 167. Note that despite the difference in the number of mappable reads, the pattern of CV against the mean remains the same. CVs are very large when the average expression is low. Individual points represent exonic regions (Flybase 5.4) cubic smoothing line fit using R's smooth.spline function.

Within each lane, for each exon, the average number of reads per nucleotide (APN) and the standard deviation were computed. The coefficient of variation (CV, standard deviation/mean) versus the APN (Figure 3) was plotted for each exon. Coverage below APN 5 is highly variable. The CV for an exon with a coverage of less than 5 reads per nucleotide is often greater than 2 and can be greater than 20. The same pattern holds for all samples examined (Figure 3), indicating that this is not an artifact of the number of mappable reads. In fact, when reads from the c167 experiment are combined and the same plot is constructed, the same pattern is seen (data not shown). Although the maximum CV is smaller for the data combined across 5 lanes, than for a single lane of data, the trend is still apparent. This, together with the results on consistency of detection, indicates that an average coverage of 5 reads is a useful target when planning an RNA-seq study. For a lane with almost 29 million mappable reads, not quite 60% of the exons in Flybase were observed at this level of coverage.

**Figure 4:**
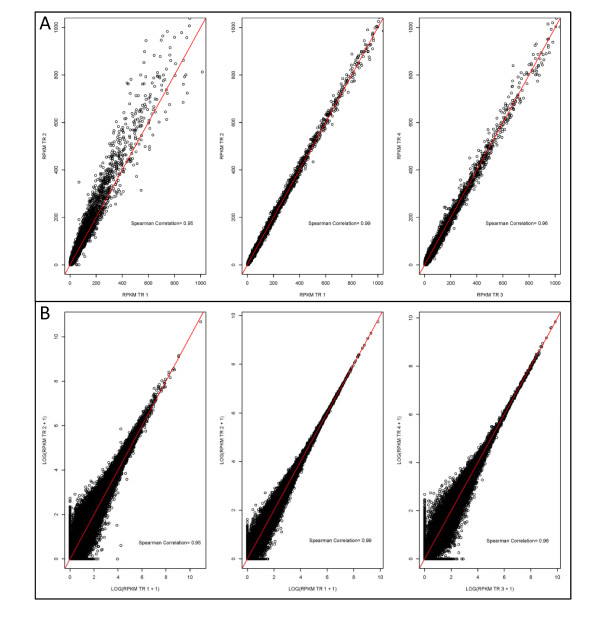
**Scatterplot of technical replicates**. Points where RPKM is 1000 or less are displayed (A). The red line is the 45 degree line. Left panel is *D. simulans *male heads BR2, middle panel is *D. melanogaster *female heads BR2 and right panel is *D. melanogaster *cell line c167 Tr3 vs TR4. Spearman correlation values are (0.95, 0.99, 0.96), respectively. Scatterplot of technical replicates on the log scale (log(RPKM+1)) for RPKM values of less than 1000) (B). The red line is the 45 degree line. Left panel is *D. simulans *male heads BR2, middle panel is *D. melanogaster *female heads BR2 and right panel is *D. melanogaster *cell line c167 Tr3 vs TR4. Spearman correlation values are (0.95, 0.99, 0.96), respectively.

There is great interest in understanding the performance of RNA-seq and to that end many studies have used the correlation coefficient as a means of assessing concordance. We plot the RPKMs of technical replicates here against each other (Figure 4A,B) to demonstrate that these plots are similar to those previously reported [15]. As expected, Pearson and Spearman correlation coefficients are very high. Spearman correlation coefficients are in excess of 0.95. However, correlation is not a measure of agreement [24-26]. By categorizing expression into several ordinal groups and then comparing two technical replicates (Table 2,3, Additional File 9), it becomes immediately obvious that there are areas of disagreement between the technical replicates, and these are not limited to exons with low coverage (Table 2,3). For one particular example given (Table 3), the Spearman correlation is 0.95 and the weighted Kappa statistic is 0.80. Although the actual agreement will depend explicitly upon the ordinal values chosen and the scale used for expression, weighted Kappa statistics for a range of possible categorizations were computed and found to be always lower than 0.9, often close to 0.6 and occasionally close to 0.5. This means that the use of RPKM as a measure of expression can be a log (or even two) different at all levels of expression. This is not sensitive to the RPKM normalization, as normalization factors [29] among technical replicates are close to 1. Indeed, this discrepancy is apparent in the simulated data, where normalization is not necessary. Sampling variance can result in differences in estimated expression of 1 log or greater. This finding may explain some of the studies which report discordance between estimates of expression from RNA-seq and known concentrations [8].

**Table 3:** Agreement between technical replicates for biological replicate 2 *D. melanogaster *female heads.

	TR2									
**TR1**	**0**	**1**	**2**	**3**	**4**	**5**	**6**	**7**	**8**	**Total**

**0**	9213	***2947***	***3***	0	0	0	0	0	0	12163

**1**	***1990***	24845	***4269***	***150***	0	0	0	0	0	31254

**2**	0	***818***	4884	***2273***	***24***	0	0	0	0	7999

**3**	0	*16*	***402***	3293	***1028***	***2***	0	0	0	4741

**4**	***1***	***2***	***2***	***172***	1669	***415***	***2***	***1***	0	2264

**5**	0	0	0	***1***	***76***	749	***213***	0	0	1039

**6**	0	0	0	0	0	***35***	357	***108***	0	500

**7**	0	0	0	0	0	0	***11***	241	***9***	261

**8**	0	0	0	0	0	0	0	***2***	54	56

**Total**	11204	28628	9560	5889	2797	1201	583	352	63	60277

RPKM was grouped into 7 categories on an approximate log10 scale. The categories were: zero reads, average less than 10, 20, 40, 80, 160, 320, 1000 and greater than 1000. Technical replicates 1 and 2 were compared. Values on the diagonal are where the ordinal categories agree and off diagonal values (bold/italic) are disagreements.

**Figure 5:**
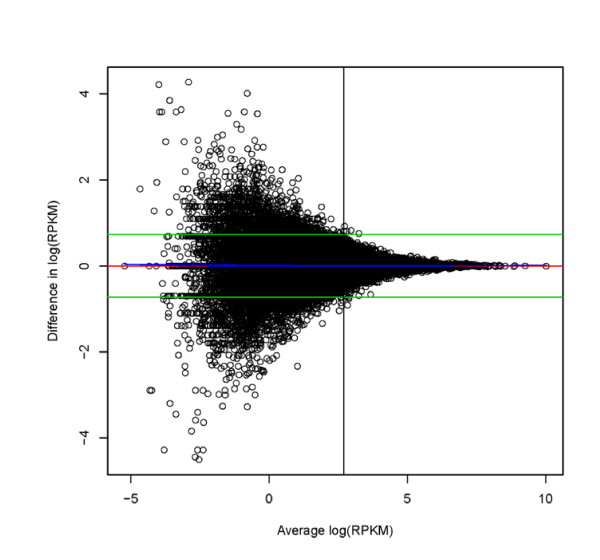
**Bland-Altman plot showing level of agreement between technical replicates for natural log transformed RPKM *D. simulans *biological replicate 3**. On the Y axis is the difference between technical replicates and on the X axis is the average between technical replicates. Green lines are the average of all differences +/- 1.96 (standard deviation of the differences). The red line is drawn at zero. The blue line is a loess fit. The discrepancy between technical replicates is a function of the estimated expression level. The horizontal line is drawn at an average coverage per nucleotide of 5. Bland-Altman plots for all the remaining comparisons among technical replicates are in Additional file 11.

The MVA plot [27], initially proposed by Bland and Altman [24,26] to assess agreement between two methods of measuring an endpoint, is an intuitive examination of agreement that helps diagnose the magnitude and functional form of disagreements. The Bland-Altman plot of the natural log-transformed data for the technical replicates of *D. simulans *biological replicate 3 (Figure 5, and Additional file 11) clearly shows that at lower levels of expression there is larger disagreement between technical replicates. However, although the absolute disagreement is a function of abundance, the moving average smoother indicates that average disagreement or bias between technical replicates is consistently linear (rather than nonlinear) over the abundance range. This is in contrast to what is observed for microarray data where a nonlinear bias (disagreement) is typically observed as a function of mean abundance making nonlinear normalization necessary [30,31]. This is also consistent with the findings of Bullard et al. (2010) [14] who find that no nonlinear normalizations are needed.

Does the choice of the Flybase definition of an exon impact the results? If the empirically defined common contig is used instead of the Flybase exon, then by definition we are only examining regions seen in all technical replicates. Common contigs show greater levels of disagreement in the estimation of amount of expression between technical replicates than the Flybase exons. Like the Flybase exons, areas of lower coverage are more likely to disagree (Additional File 9). The disagreement across technical replicates decreases as the coverage increases for all the data examined here (Table 2, Additional file 9).

Consistent with other reports [14,30] biological variation is larger than technical variation. The number of exons detected in both replicates is lower between biological replicates (Table 2). Some of these differences may be due to the flow cell/lane assignments, which are unknown for all of these experiments. Consistent with previous reports, the level of coverage makes a large difference and some of the biggest disagreements are in the *D. simulans *experiments where 1 biological replicate has ~5 million reads where the other biological replicates had ~28 million reads. These samples must have been run not only on different flow cells but also on different iterations of the technology. Based on previous findings, the finding that these samples have the largest differences and lowest agreement is not surprising.

## Discussion

The number of exons detected, was approximately 64% (out of 60,277 exons covering genome positions total) in lanes with 5-7 million reads and 84% in lanes with approximately 27 million reads. The results of this study indicate exons are not consistently detected among technical replicates when the average coverage for that exon is less than 5 reads per nucleotide. Additionally, the coefficient of variation can be very high for exons with coverage of less than 5 reads per nucleotide. Between lanes, exons that are detected in both lanes with one of those being at low coverage, disagree in the estimates of abundance (RPKM) between technical replicates, leading us to conclude that when analyzing data for differential expression, one could consider identifying exons with coverage less than 5 for closer examination. These findings are consistent across different versions of the technology, different species, and different laboratories (E1 and E2 vs E3). The number of exons covered with an average of 5 reads per nucleotide--only 21% of all exons in lanes with 5-7 million reads and 58-60% in lanes with 27 million reads--is substantially smaller than the number of exons detected.

Although agreement improves once coverage is above 5, it is not perfect and disagreement can be large even when coverage is high. Why are there such large discrepancies in detection and in estimates of expression across technical replicates? The underlying assumption for all gene expression technology, whether microarray, SAGE, Q-PCR or RNA-seq, is that the final measurement of gene expression for a particular sample is proportional to the underlying population of mRNA extracted from that sample. The variability in exon detection, and the disagreements in estimates of expression are perplexing. A possible explanation would be the choice of the particular normalization. However, the results are virtually identical if we use an average per nucleotide or RPKM. A normalization constant [29] was also estimated and found to be close to 1 among technical replicates, indicating that these results cannot be explained by improper normalization. There are at least two obvious explanations for observing this disagreement: insufficient mixing and a very low sampling fraction.

If any of the solutions generated during preparation for cluster generation are insufficiently mixed, then the distribution of reads across samples is expected to be uneven. This would be similar to what Student saw in his experiment with yeast cells [32]. In this experiment, insufficient mixing was partially attributed to the tendency of yeast cells to "stick together in groups which was not altogether abolished even by vigorous shaking." Student developed the negative binomial in response to this observation. In the negative binomial distribution the variance is larger than in a Poisson distribution. The standard Poisson has a variance equal to the mean. Poisson distributions that allow for over dispersion are general, and the negative binomial is one particular type of over dispersion. The overdispersed Poisson has been proposed for modeling RNA-seq data [12,14,15,33,34].

For both the *D. melanogaster *and *D. simulans *experiments, technical replicates were derived from an individual library analyzed on multiple lanes. Thus, discordance between the technical replicates must have occurred after library synthesis, presumably during generation of the templates on the flow cell. We can envision other experimental plans in which technical replicates are derived from an individual sample, prior to library synthesis. In this latter case, discordance between technical replicates could arise during or after library preparation. Modifications of the protocol have been developed that omit potential bias due to amplification [35]. An inspection of the cluster generation protocol (Figure 2), carried out after library synthesis, reveals additional places where variation might be introduced. The initial step of the protocol entails at least one large dilution step. Any uneven distribution of molecules in the library at this point, whether due to incomplete mixing or DNA-DNA interactions assisted by cations [36], could lead to differences in the molecules loaded onto a lane. Single stranded libraries may have better mixing. If there is aggregation of molecules in solution, then the current protocol could be modified to a dilution series with particular care taken to ensure molecules are mixed well and neither aggregate nor degrade in the process. Non-uniformity in solution is not limited to cDNA given that RNA molecules, used as input into library synthesis, can form intra- and intermolecular interactions. RNA solutions containing high concentrations of divalent metal ions are particularly prone to forming RNA aggregates [37]. As an extreme example, more than half of the RNA in a pool of random RNA sequences was found to aggregate together through self-complementarity and precipitate out of solution under conditions favoring intramolecular interactions [38]. Although it is not possible to determine from these experiments if unequal mixing is a problem, removing or reducing the potential for this problem should be a relatively straightforward modification of the current protocols.

**Figure 6:**
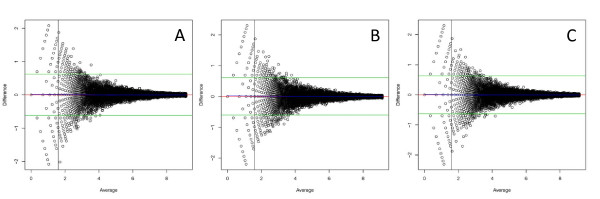
**Bland-Altman plot for simulated data**. The data were log transformed and the average of the two technical replicates is on the X axis and the difference between technical replicates is on the Y axis. (A) Simulated replicates 1 versus 2. (B) Simulated replicates 1 versus 3. (C) Simulated replicates 2 versus 3. Green lines are the average of all differences +/- 1.96 (standard deviation of the differences). The red line is drawn at zero. The blue line is a lowess fit.

The other possibility is that discrepancies among technical replicates are due to the sampling fraction, 0.0013% for 30 million reads (Figure 2). This fraction is so low that the behavior of random sampling in this particular scenario should be examined. The simulation study performed here indicates that data are consistent with this hypothesis. Marioni (2008) [15] also reported data consistent with randomness. Agreement between technical replicates in the simulated data (Figure 6A,B,C) shows the same patterns as the three sets of real data presented here. In the simulated data, there are also examples of genes with substantial disagreement among technical replicates. This indicates that the observed disagreement can be explained by the low sampling fraction, and that the results are consistent with the expectations of random variation. What is initially surprising is the size of the random fluctuation. However, when the sampling fraction is considered, the results are less surprising.

This study demonstrates that the current nature of the technology is to expect variation between single lanes both in exon detection and estimation of gene expression using RPKM. Technical variation, while smaller than the biological variation, cannot be ignored and should be accounted for in the study design.

The fundamental principles of statistical experimental design are thus just as important as ever, even with modern technology. R.A. Fisher defined these fundamentals to be randomization, replication and blocking [13,39]. The optimal experimental design strategy will depend on the objectives of the study at hand. Randomization and blocking are important to incorporate in order to avoid confounding of biological with systematic experimental effects. Likely experimental effects in mRNA Seq data include lane, flow cell or library preparation batch. Randomized block designs have been shown to be an efficient use of resources while ensuring legitimate comparisons to be made. Multiplex designs [12] represent another good solution which would ensure more complete coverage of exons across biological replicates and eliminate lane as a potential confounding variable in small experiments. Consider the following simple example of three biological replicates for treatments and three biological replicates for controls for a Drosophila experiment. If each sample is run on a single lane and more coverage is needed, then another full set of 6 lanes is necessary. In contrast, a single multiplexed run where all 6 samples are run on all 6 lanes has been proposed [12]. Exons seen in some technical replicates but not others are clearly a result of technical variation. In this design the total coverage remains almost the same as in the simple design. The added benefit is that small numbers of extra lanes can be run if more coverage is desired.

A replication strategy must be chosen as well with an appropriate balance between biological replicates and technical replicates [40-43]. While, increasing the number of biological replicates increases the precision and generalizability of a study more than increasing the number of technical replicates; for studies where low abundant mRNAs are the focus, increasing technical replication may also be important.

## Conclusions

RNA-seq experiments need to replicate the results both technologically and biologically as the technical variation in exon presence and absence as well as amount of coverage is not negligible. Consistent with random variation due to low sampling fractions, technical variation is most pronounced in the variability of detection of exons when coverage is low. However, disagreements between estimates of expression can occur at all levels of coverage.

## Authors' contributions

LMM designed the study, did analyses and wrote the paper, KKL did analyses, designed and executed simulation study, contributed to writing of the paper, AMM designed experiments and writing of the paper, VA carried out all bioinformatics pipeline design and execution, ALO designed experiments and contributed to the paper, LJY designed experiments and contributed the paper, and SVN designed experiments and contributed to writing of the paper. All authors read and approved the final manuscript.

## Supplementary Material

Additional file 1 **Overlapping exons combined into single genomic region**. D. melanogaster ovo gene (Flybase ID BFgn0003028) used as an example of combining overlapping exons into a single genome region for mapping purposes. Format PDF. View with Adobe. Click here for file

Additional file 2 **Chromosome postions for overlapping exons combined into single genomic regions**. Format TSV. View with Wordpad. Click here for file

Additional file 3 **Perl script for mapping between exons and a genome region**. Format PL. View with Wordpad. Click here for file

Additonal File 4 **Perl script that converts contiguous sequences in a SAMtools consensus pileup to FASTA, BED, or coordinate tables**. Format PL. View with Wordpad. Click here for file

Additional file 5 **Perl script to find common sequences from contig BEDs**. Format PL. View with Wordpad. Click here for file

Additional file 6 **St_2a_D.melanogaster_common_contigs.bed**. BED file containing chromosome postion, start, end and common_contig_ID for *D. melanogaster *experiment (Experiment 1). Format BED. View with Wordpad. Click here for file

Additional file 7 **St_2b_Dsimulans_common_contigs.bed**. BED file containing chromosome postion, start, end and common_contig_ID for *D. simulans *(Experiment 2). Format BED. View with Wordpad. Click here for file

Additional file 8 **St_2c_167_common_contigs.bed. BED file containing chromosome postion, start, end and common_contig_ID for c167 experiment (Experiment 3)**. Format BED. View with Wordpad. Click here for file

Additional file 9 **Agreement between technical replicates and biological replicates for RPKM measured on common contigs**. A comparison in the agreement of the estimation of the amount of expression. Agreement in whether common contigs are expressed, expressed at a low level (0 < RPKM < 20), and expressed at a high level (RPKM > 20) and agreement on a 9 level ordinal scale as follows: RPKM less than 10, 20, 40, 80, 160, 320, 1000 and greater than 1000. Agreement was measured using a kappa coefficient. The number of common contigs where disagreement is greater than 2 logs and greater than one log are also given. Format XLS. View with Excel. Click here for file

Additional file 10 **Length of the Exon does not explain disagreement in technical replicates**. Coverage plots of the c167 cell lines data. The Y axis is the average coverage across all technical replicates. A bar is drawn if the exon is present (at any coverage level) in that technical replicate. The 10th percentile represents that bottom 10% of the exons in length while the 90th percentile represents the top 10% of exons by length. Format PDF. View with Adobe Click here for file

Additional file 11 **Bland Altman plots for each biological replicate**. Click here for file
