# Supplementary material for: RNA-seq: technical variability and sampling
Source: BMC Genomics. 2011 Jun 6;12:293. doi: 10.1186/1471-2164-12-293 (PMC3141664; doi:10.1186/1471-2164-12-293)

Supplementary Figure 1. Overlapping exons combined into single genomic region. D. melanogaster ovo gene (Flybase ID BFgn0003028) used as an example of combining overlapping exons into a single genome region for mapping purposes.

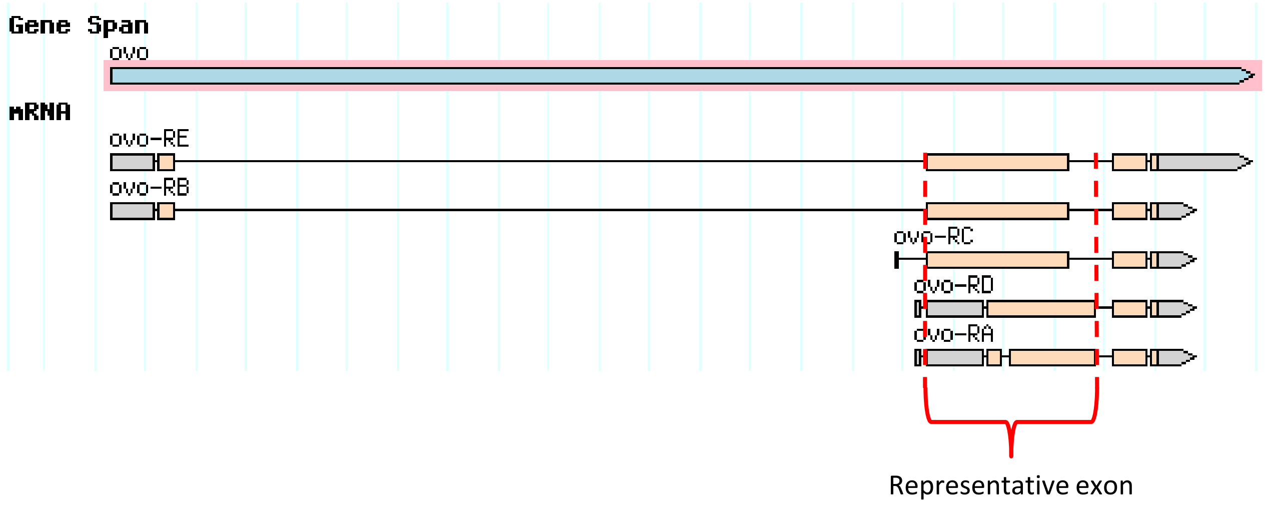

Supplement: Additional file 1 — Overlapping exons combined into single genomic region. D. melanogaster ovo gene (Flybase ID BFgn0003028) used as an example of combining overlapping exons into a single genome region for mapping purposes. Format PDF. View with Adobe. [file 1471-2164-12-293-S1.PDF]
