# Supplementary material for: RNA-seq: technical variability and sampling
Source: BMC Genomics. 2011 Jun 6;12:293. doi: 10.1186/1471-2164-12-293 (PMC3141664; doi:10.1186/1471-2164-12-293)
Supplement: Additional file 10 — Length of the Exon does not explain disagreement in technical replicates. Coverage plots of the c167 cell lines data. The Y axis is the average coverage across all technical replicates. A bar is drawn if the exon is present (at any coverage level) in that technical replicate. The 10th percentile represents that bottom 10% of the exons in length while the 90th percentile represents the top 10% of exons by length. Format PDF. View with Adobe [file 1471-2164-12-293-S10.PDF]

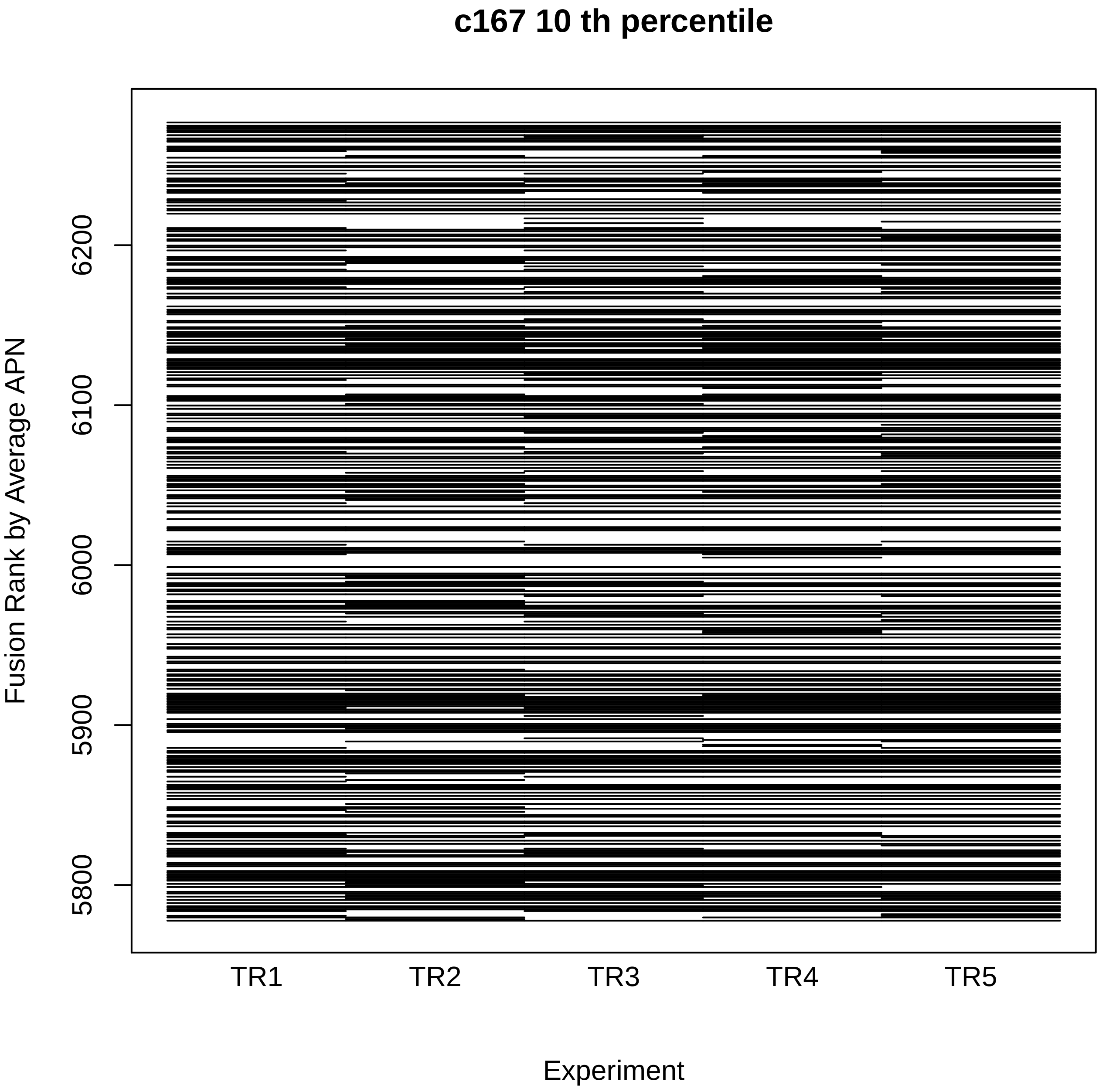

c167 10 th percentile
Fusion Rank by Average APN
6200
6100
6000
5900
5800
TR1
TR2
TR3
TR4
TR5
Experiment

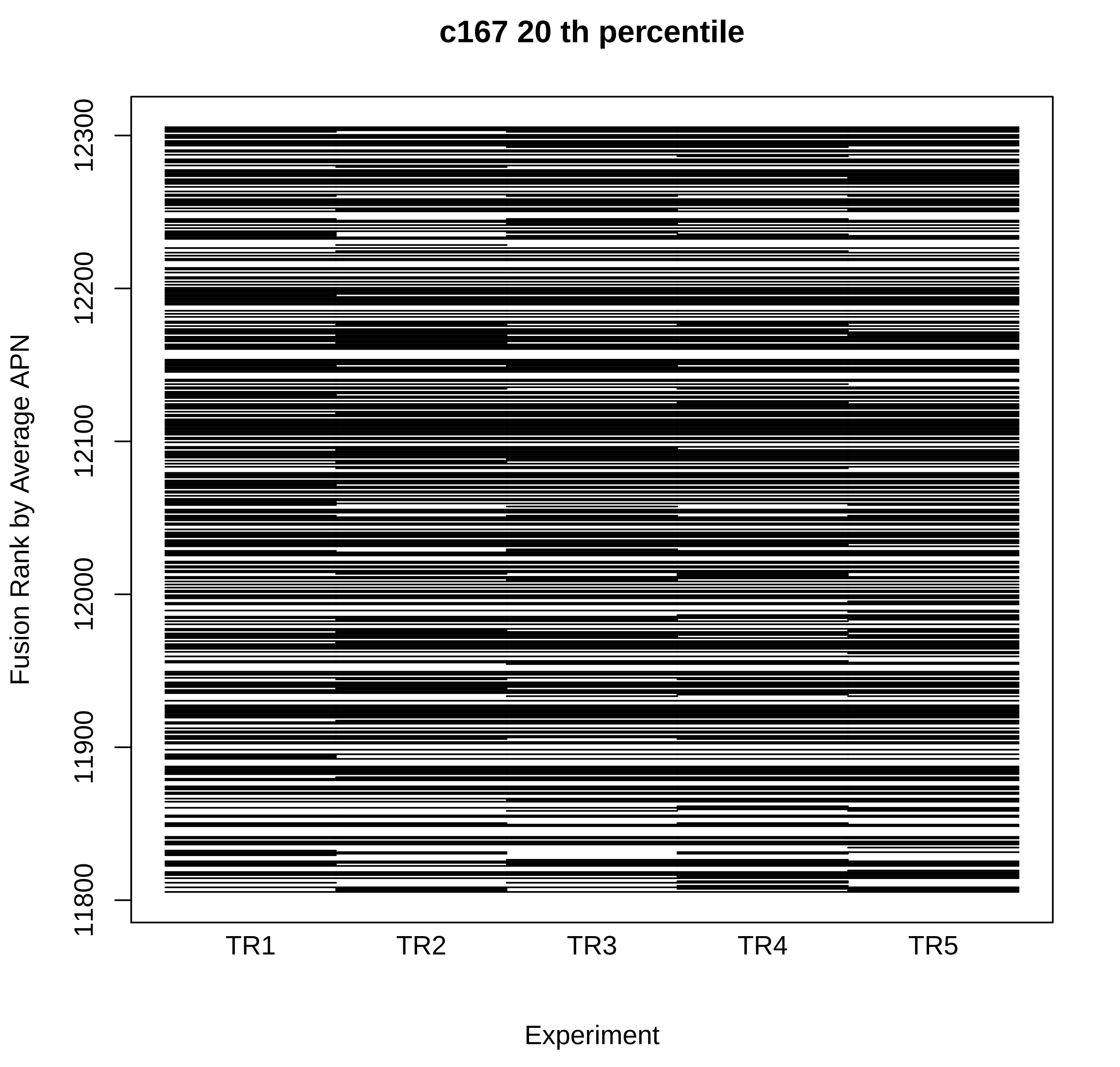
c167 20 th percentile
Fusion Rank by Average APN
12300
12200
12100
12000
11900
11800
TR1
TR2
TR3
TR4
TR5
Experiment

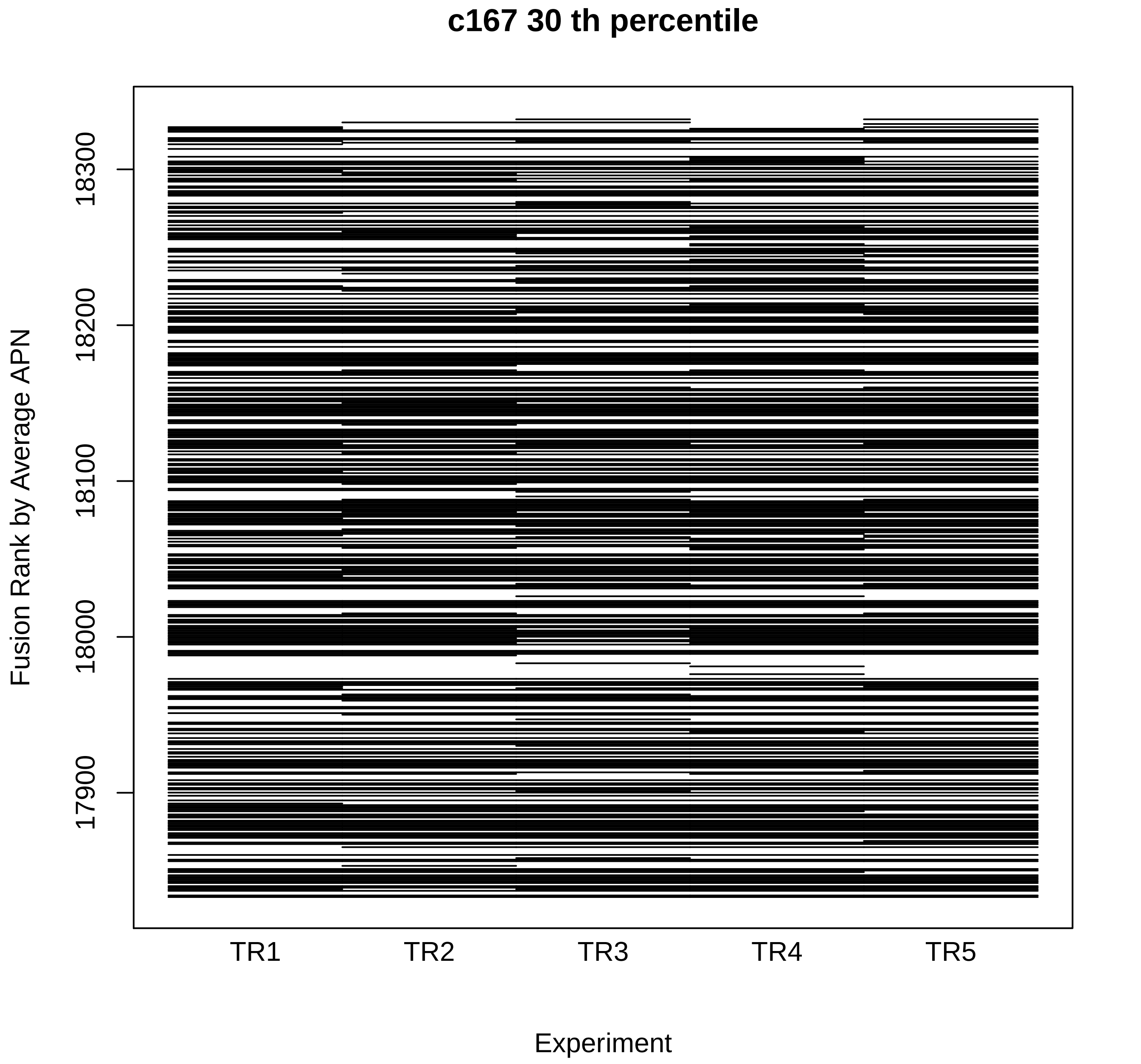

c167 30 th percentile
Fusion Rank by Average APN
18300
18200
18100
18000
17900
TR1
TR2
TR3
TR4
TR5
Experiment

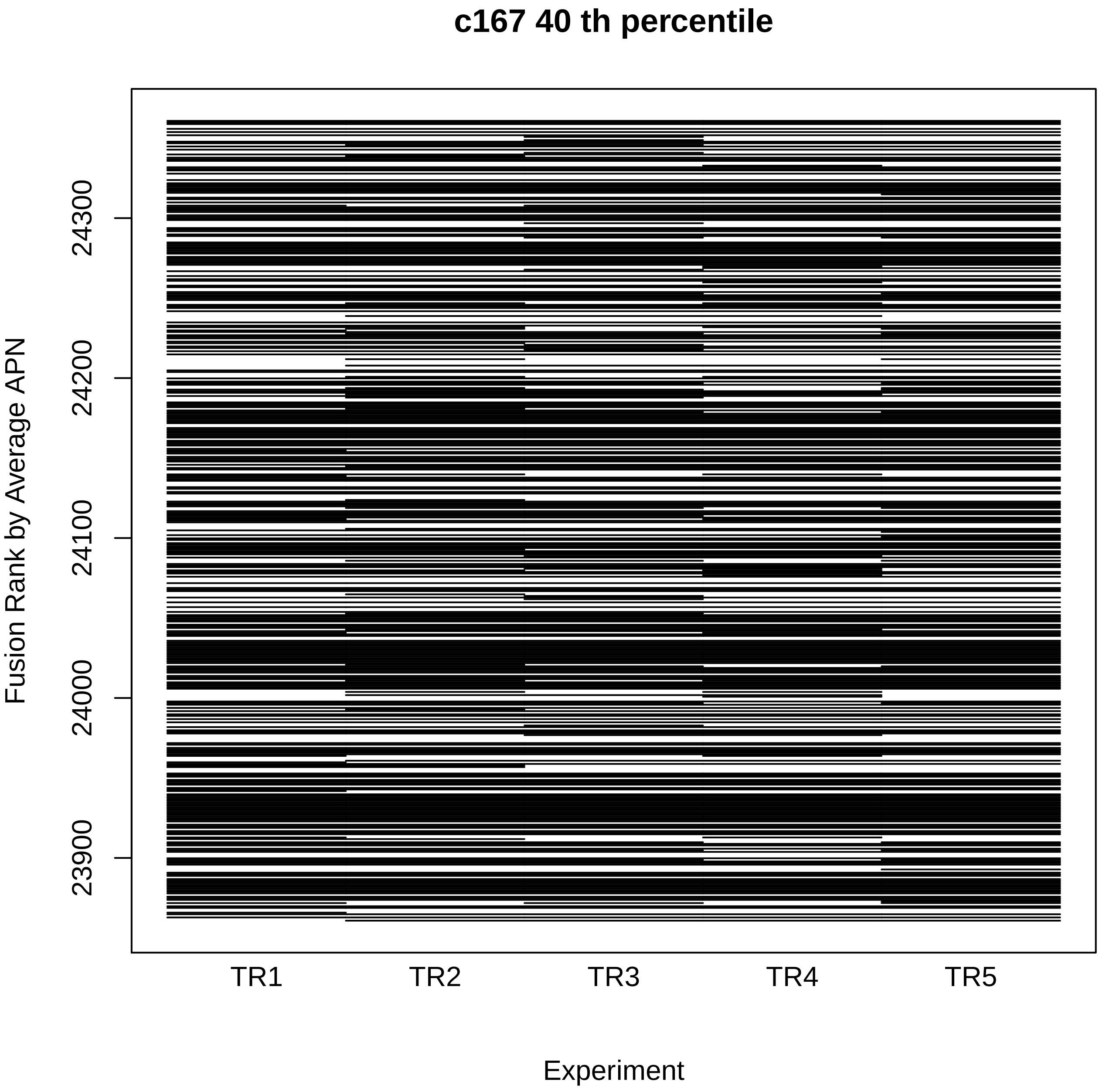

c167 40 th percentile
Fusion Rank by Average APN
23900
24000
24100
24200
24300
TR1
TR2
TR3
TR4
TR5
Experiment

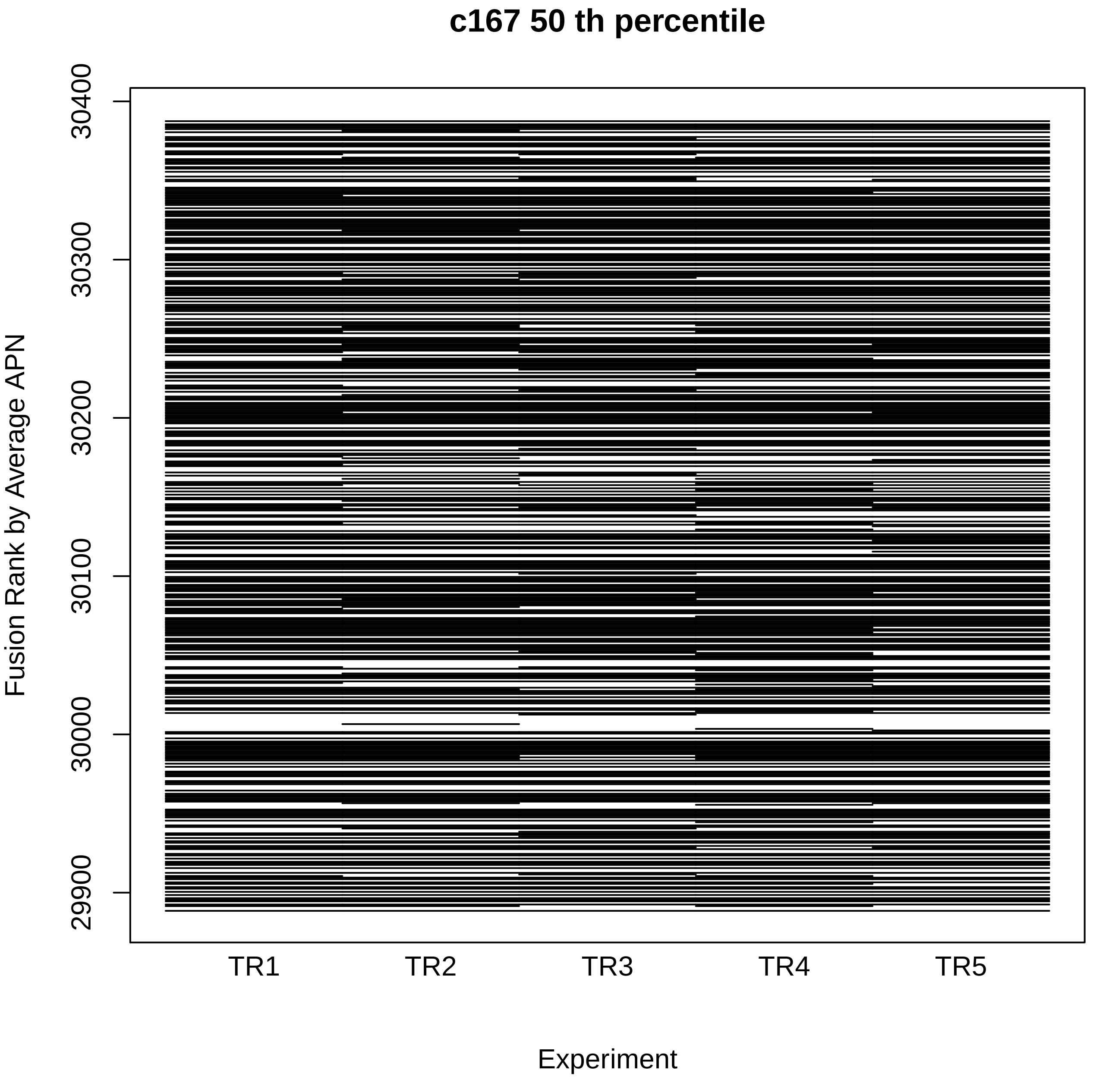
c167 50 th percentile
Fusion Rank by Average APN
30400
30300
30200
30100
30000
29900
TR1
TR2
TR3
TR4
TR5
Experiment

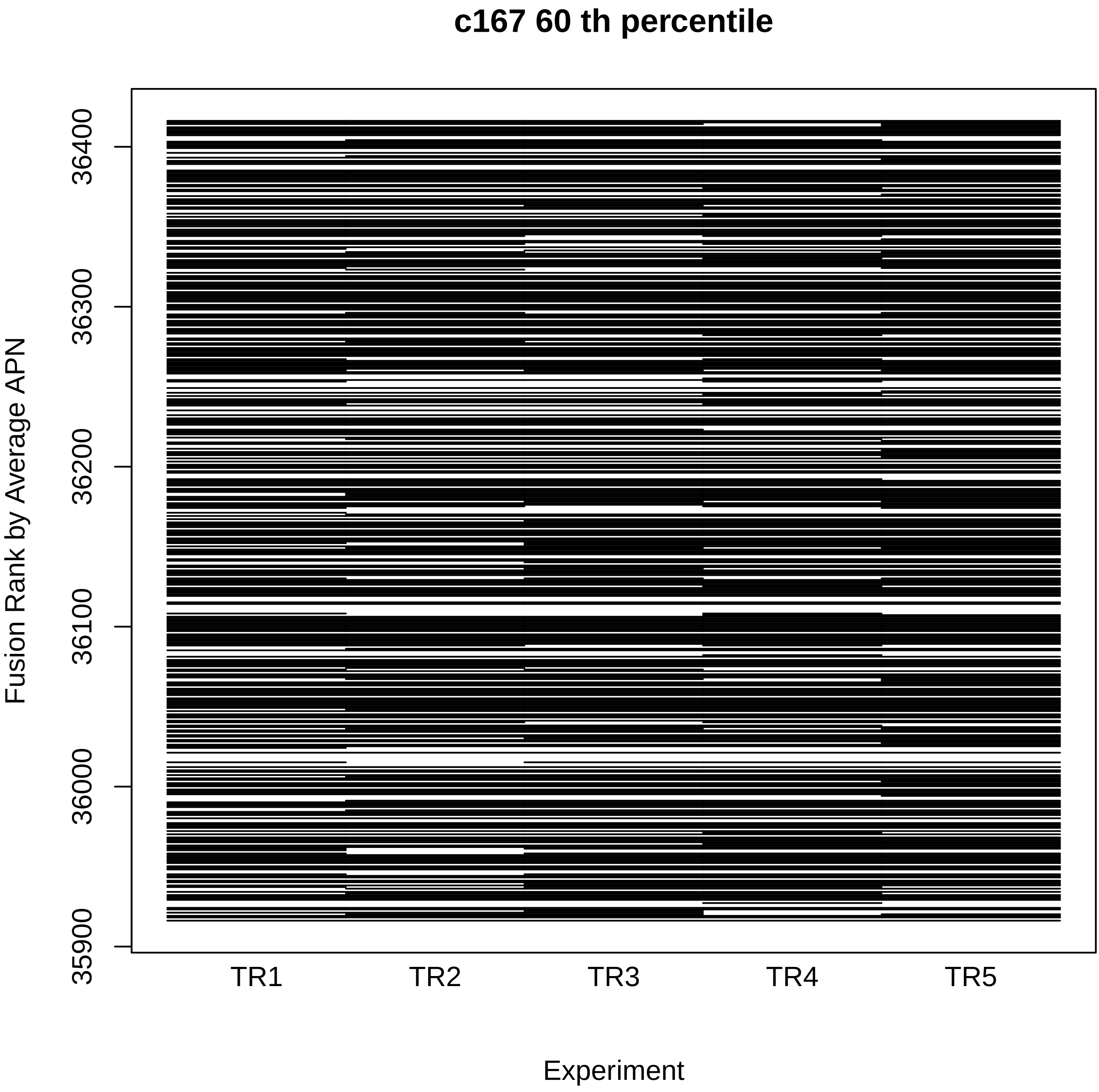

c167 60 th percentile
Fusion Rank by Average APN
35900
36000
36100
36200
36300
36400
TR1
TR2
TR3
TR4
TR5
Experiment

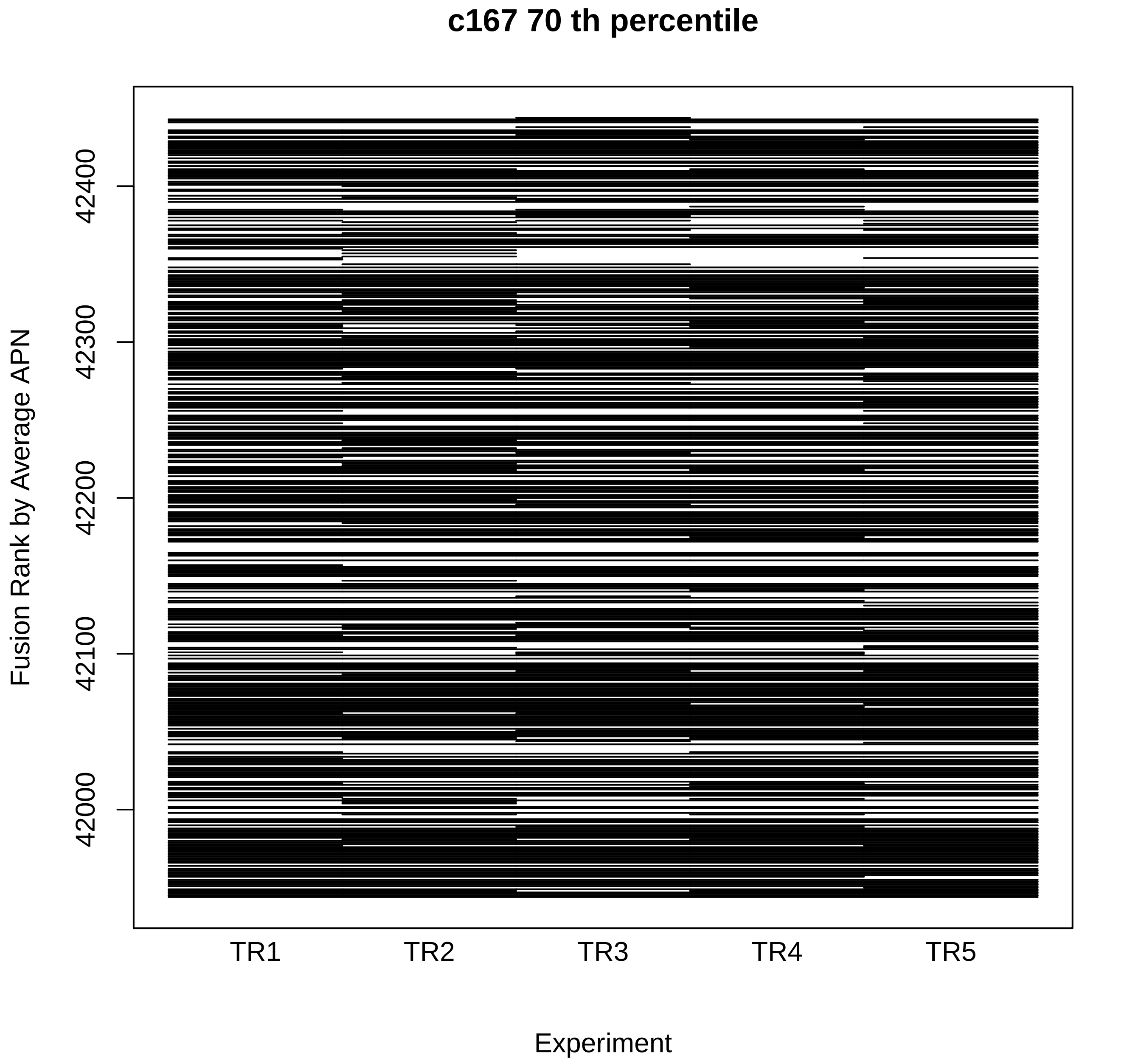

c167 70 th percentile
Fusion Rank by Average APN
42000
42100
42200
42300
42400
TR1
TR2
TR3
TR4
TR5
Experiment

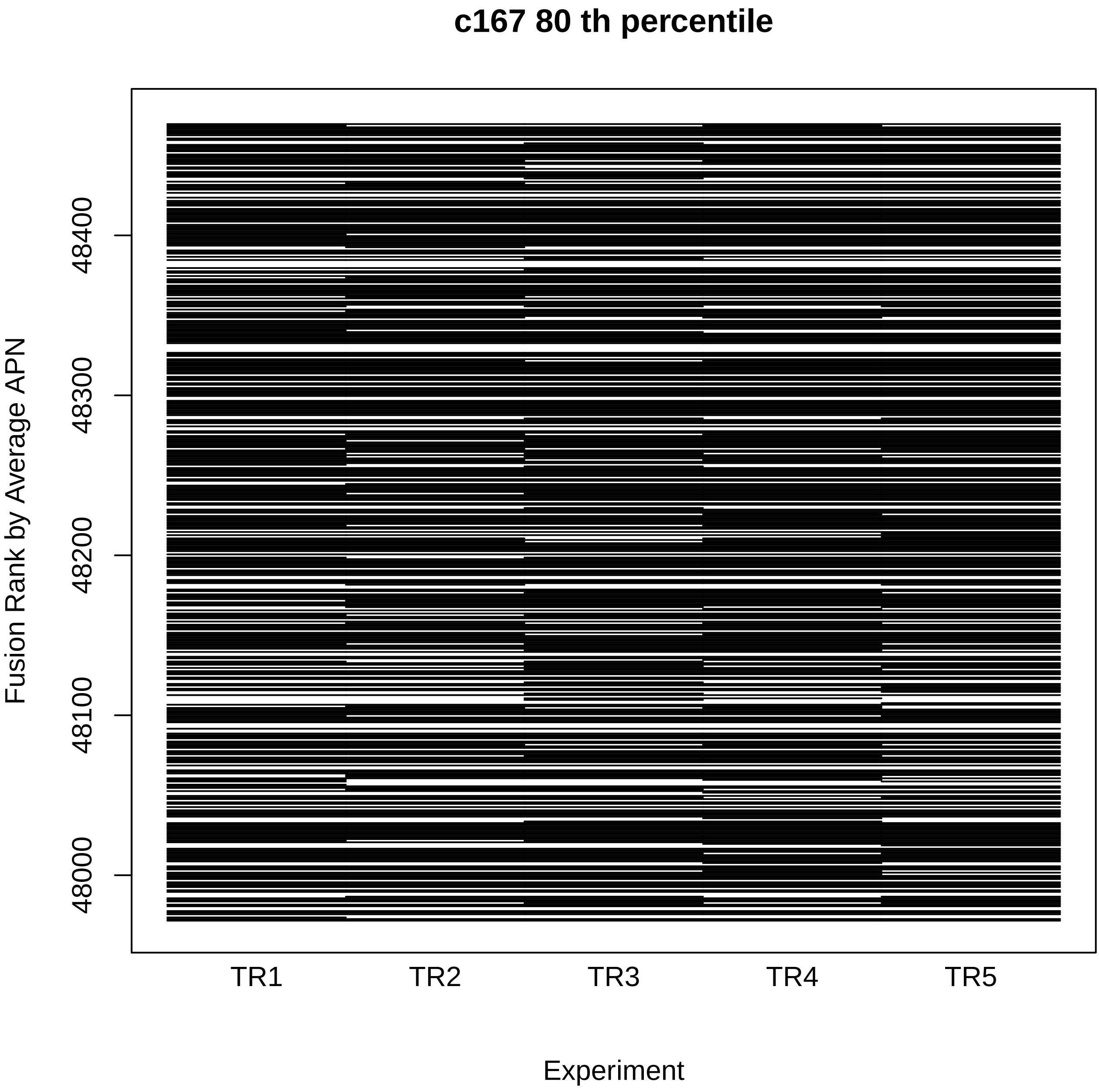
c167 80 th percentile
Fusion Rank by Average APN
48000
48100
48200
48300
48400
TR1
TR2
TR3
TR4
TR5
Experiment

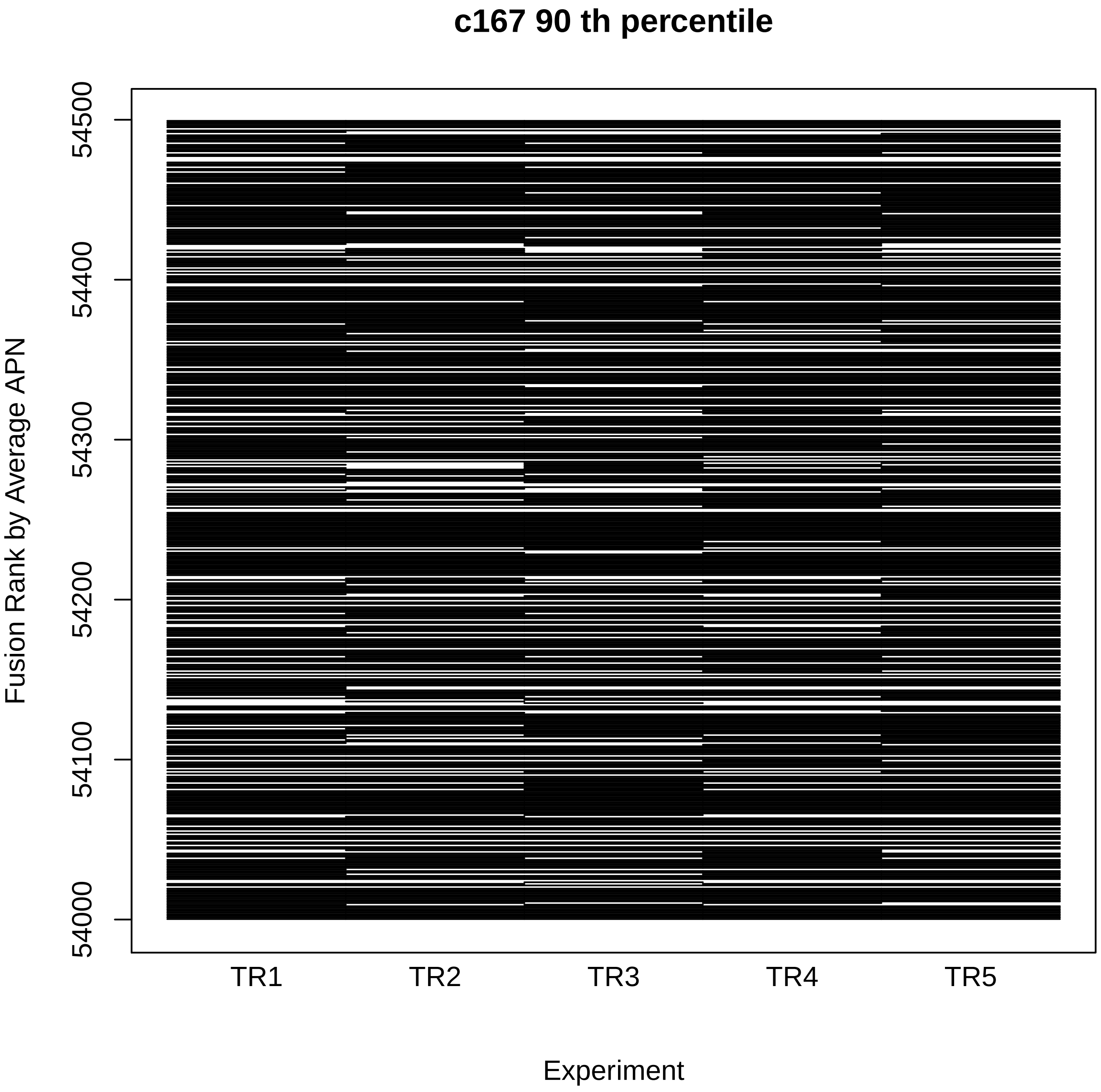

c167 90 th percentile
Fusion Rank by Average APN
54000
54100
54200
54300
54400
54500
TR1
TR2
TR3
TR4
TR5
Experiment
