# Supplementary material for: RNA-seq: technical variability and sampling
Source: BMC Genomics. 2011 Jun 6;12:293. doi: 10.1186/1471-2164-12-293 (PMC3141664; doi:10.1186/1471-2164-12-293)
Supplement: Additional file 11 — Bland Altman plots for each biological replicate. [file 1471-2164-12-293-S11.PDF]

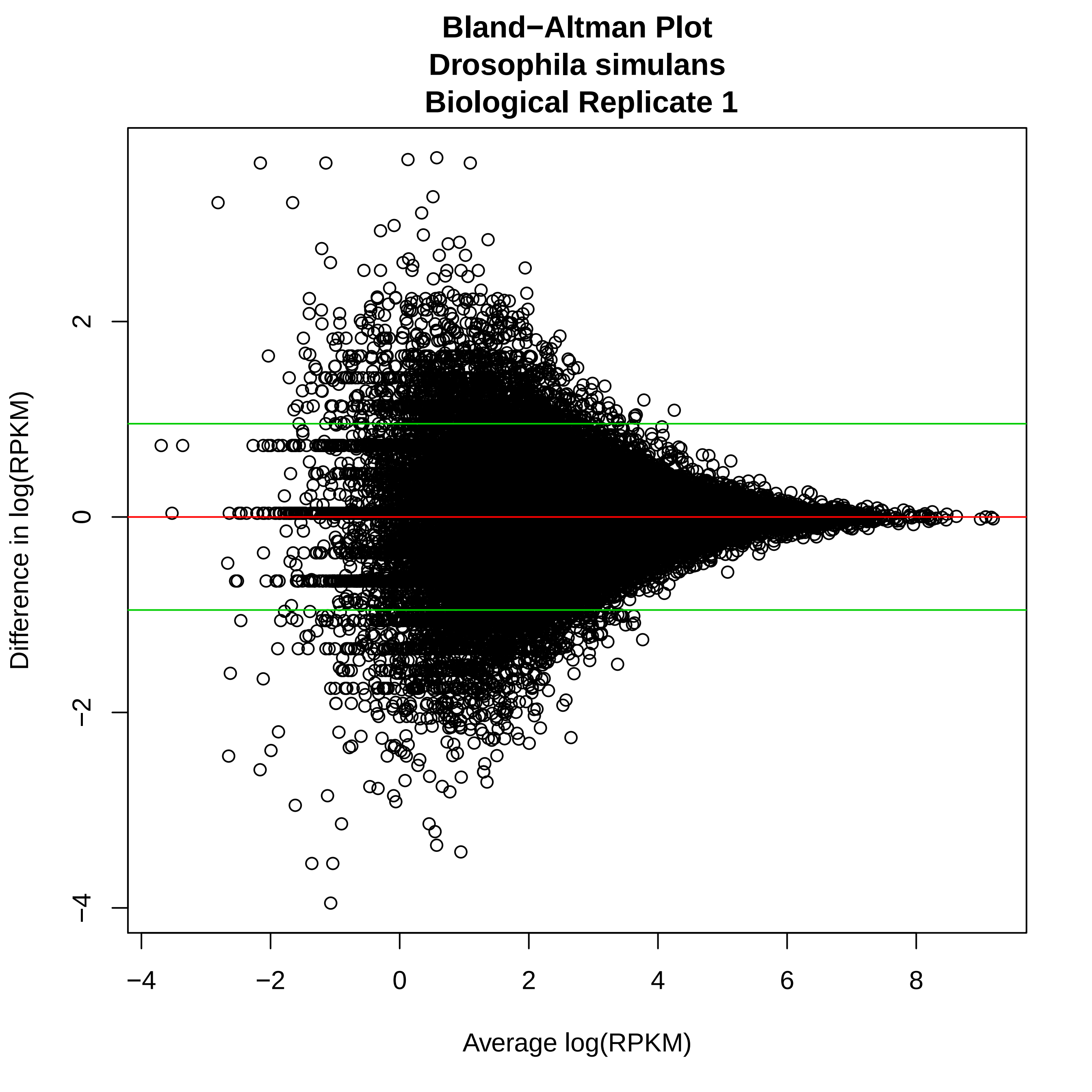
Bland−Altman Plot
Drosophila simulans
Biological Replicate 1
Difference in log(RPKM)
Average log(RPKM)
−4
−2
0
2
4
6
8

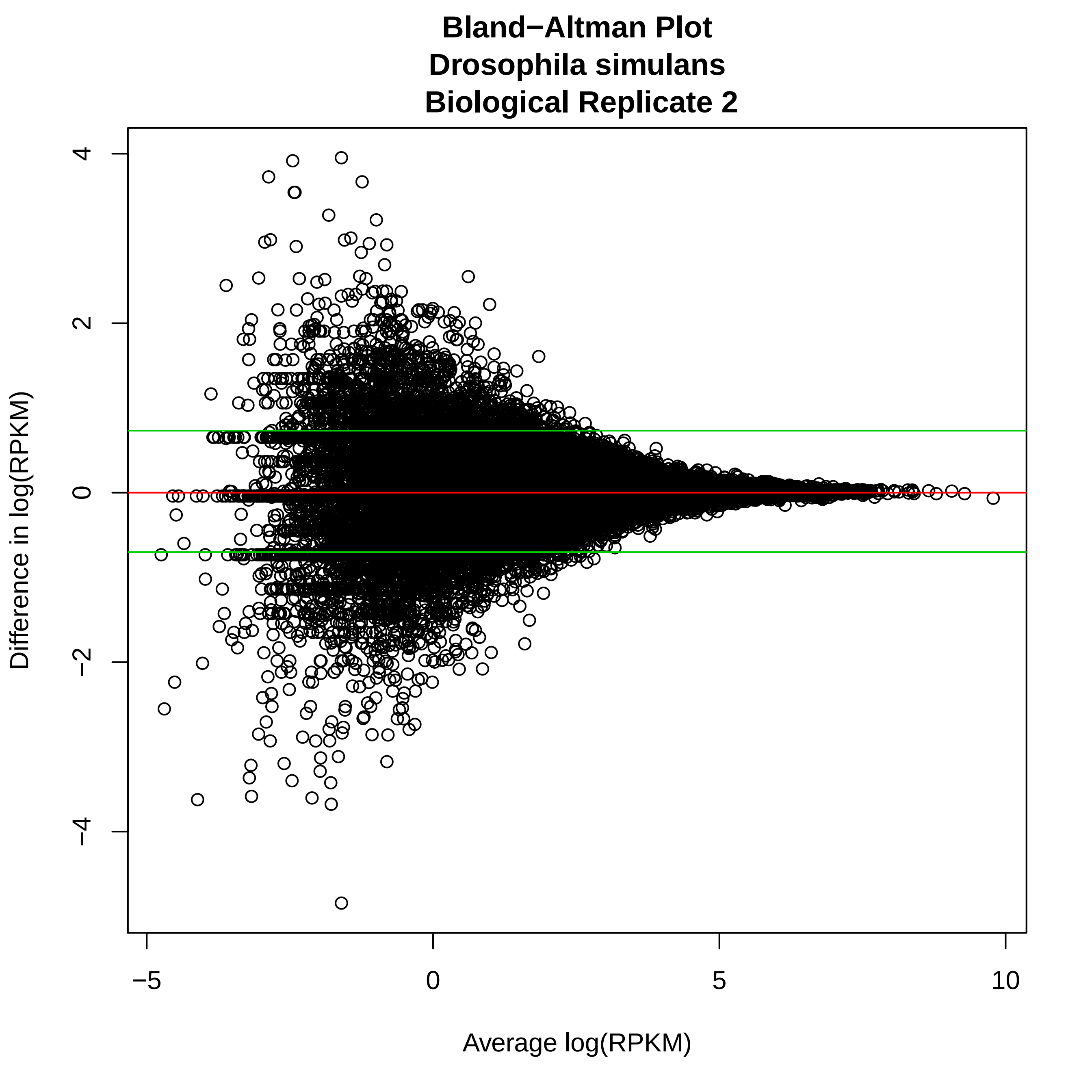
Bland−Altman Plot
Drosophila simulans
Biological Replicate 2
Difference in log(RPKM)
Average log(RPKM)
4
2
0
−2
−4
−5
0
5
10

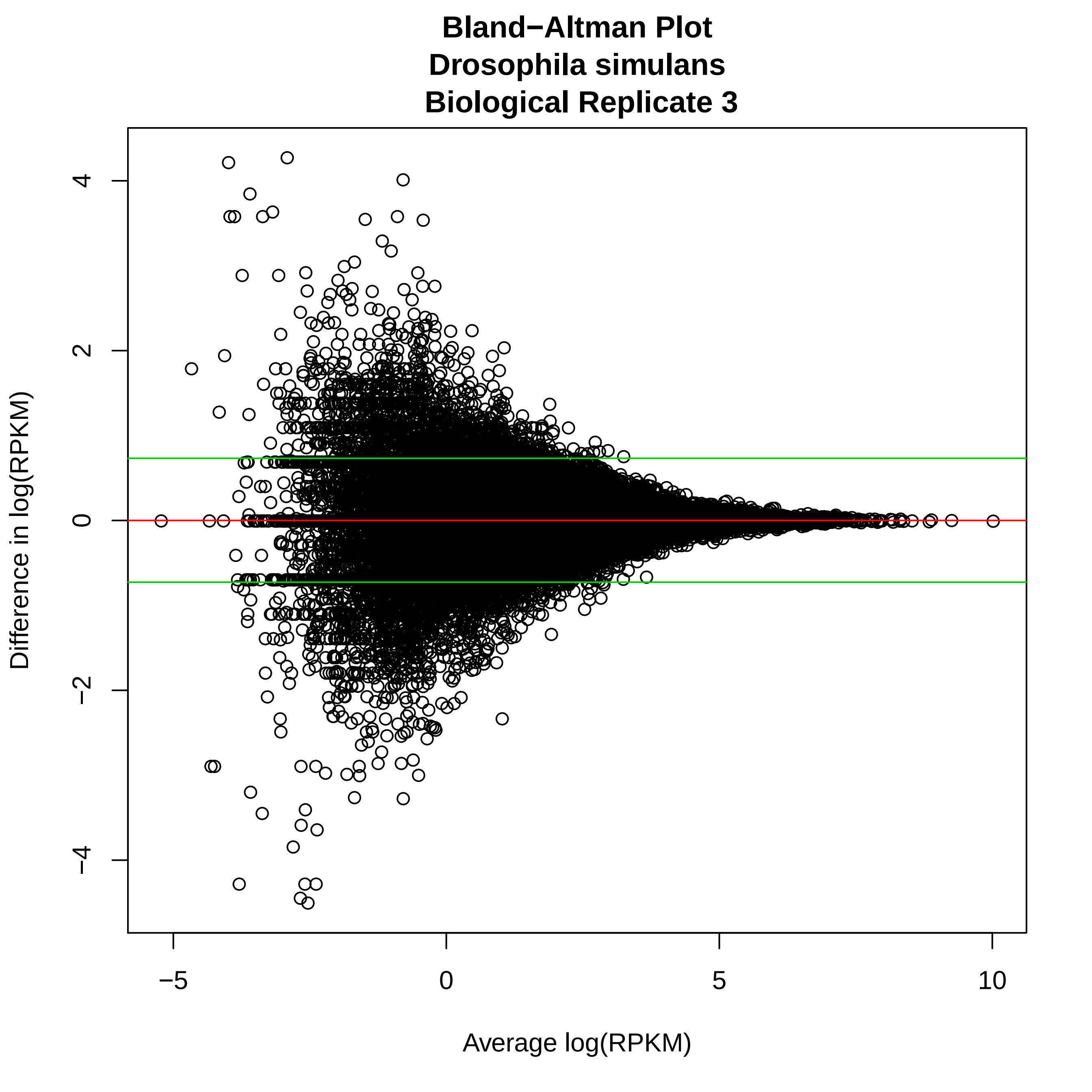

Bland−Altman Plot
Drosophila simulans
Biological Replicate 3
Difference in log(RPKM)
Average log(RPKM)
4
2
0
−2
−4
−5
0
5
10

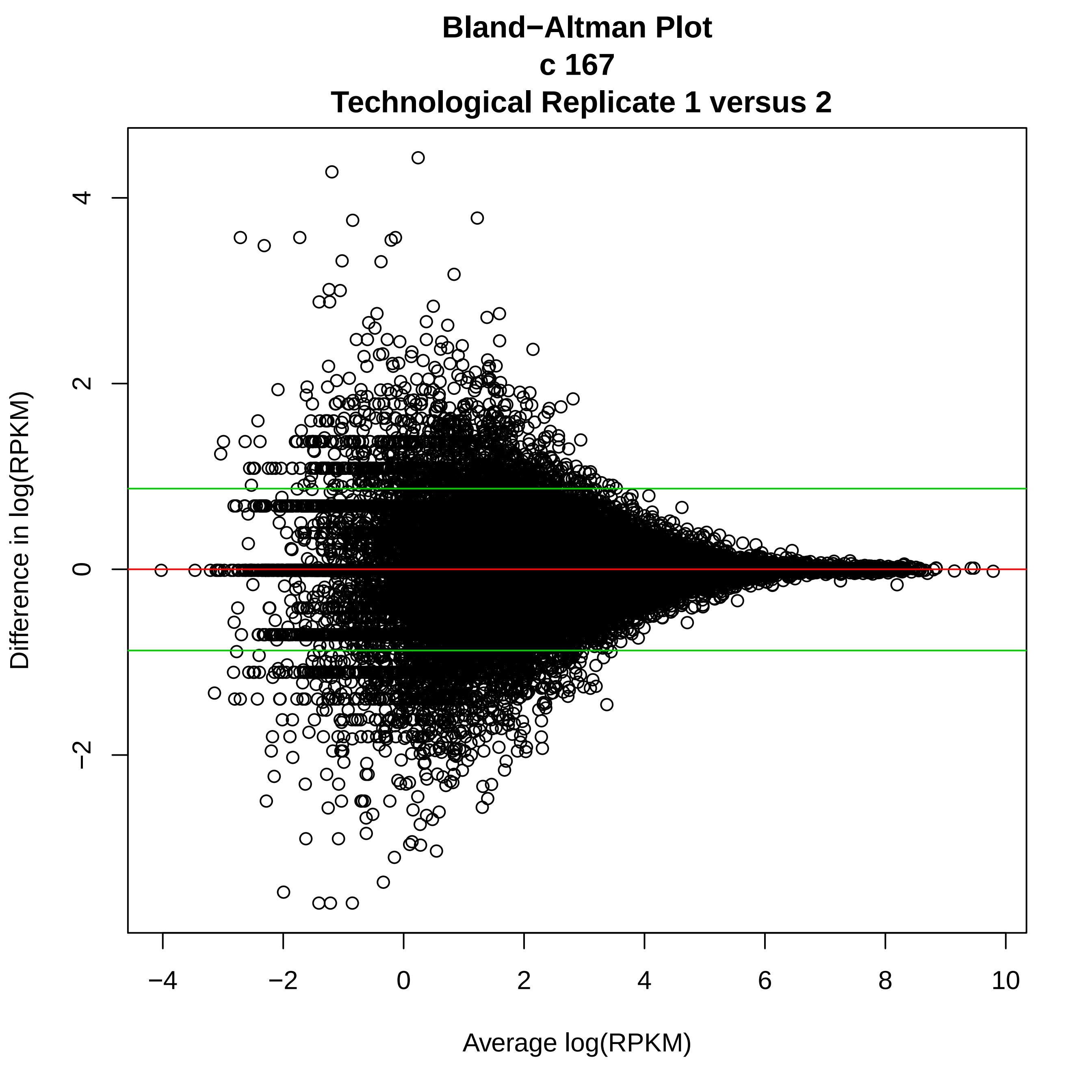
Bland−Altman Plot
c 167
Technological Replicate 1 versus 2
Difference in log(RPKM)
Average log(RPKM)
−2
0
2
4
−4
−2
0
2
4
6
8
10

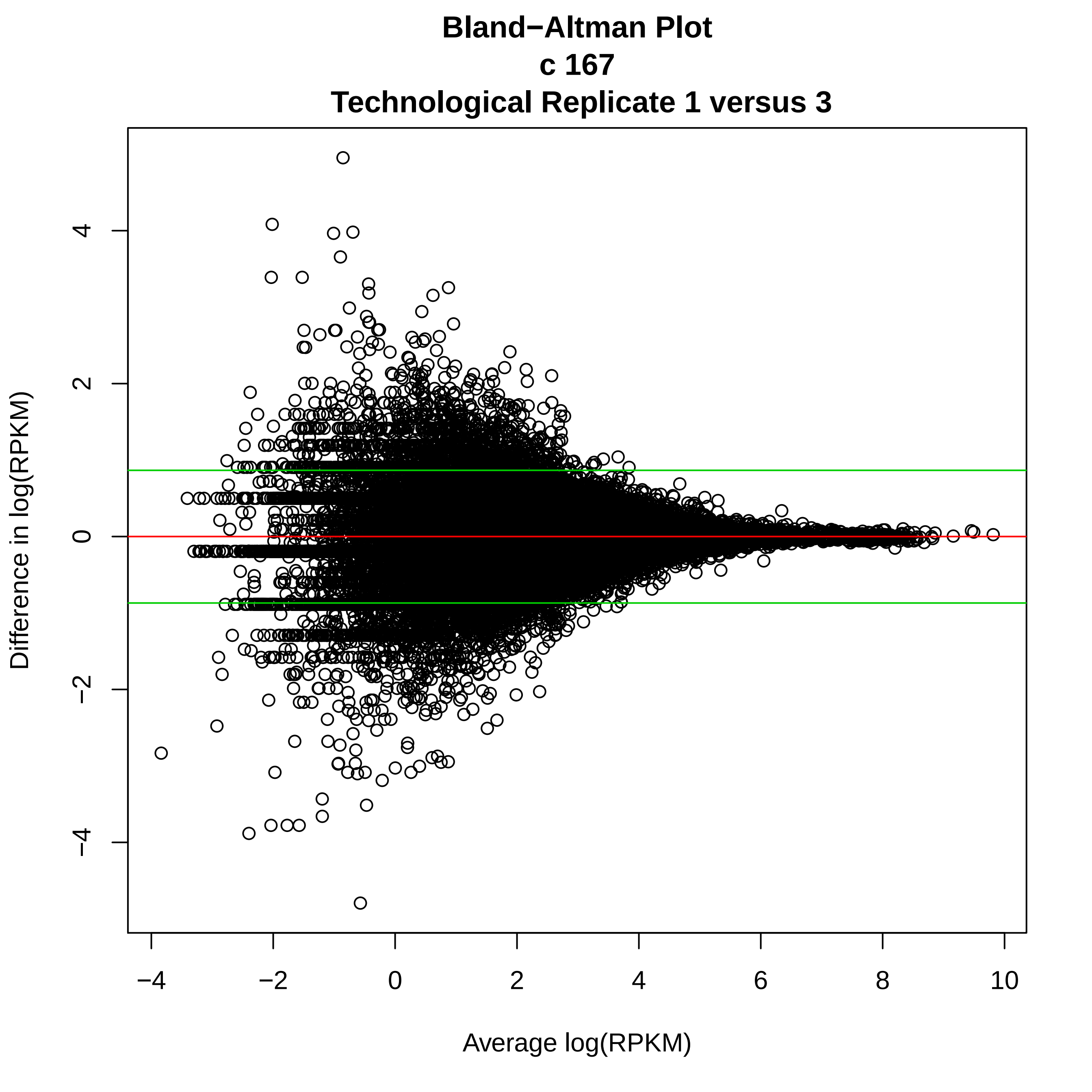
Bland−Altman Plot
c 167
Technological Replicate 1 versus 3
Difference in log(RPKM)
Average log(RPKM)
−4
−2
0
2
4
6
8
10

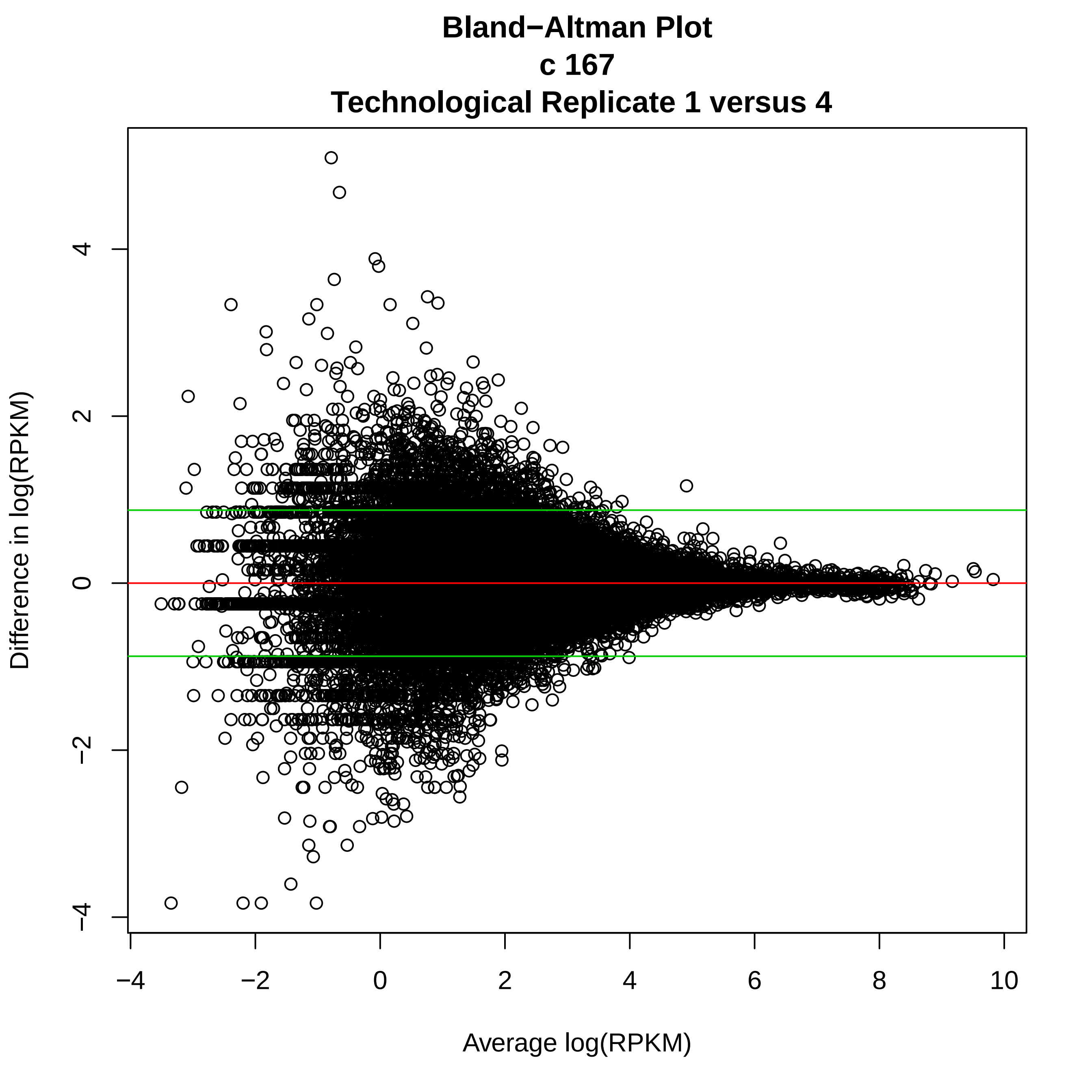
Bland−Altman Plot
c 167
Technological Replicate 1 versus 4
Difference in log(RPKM)
Average log(RPKM)
−4
−2
0
2
4
6
8
10

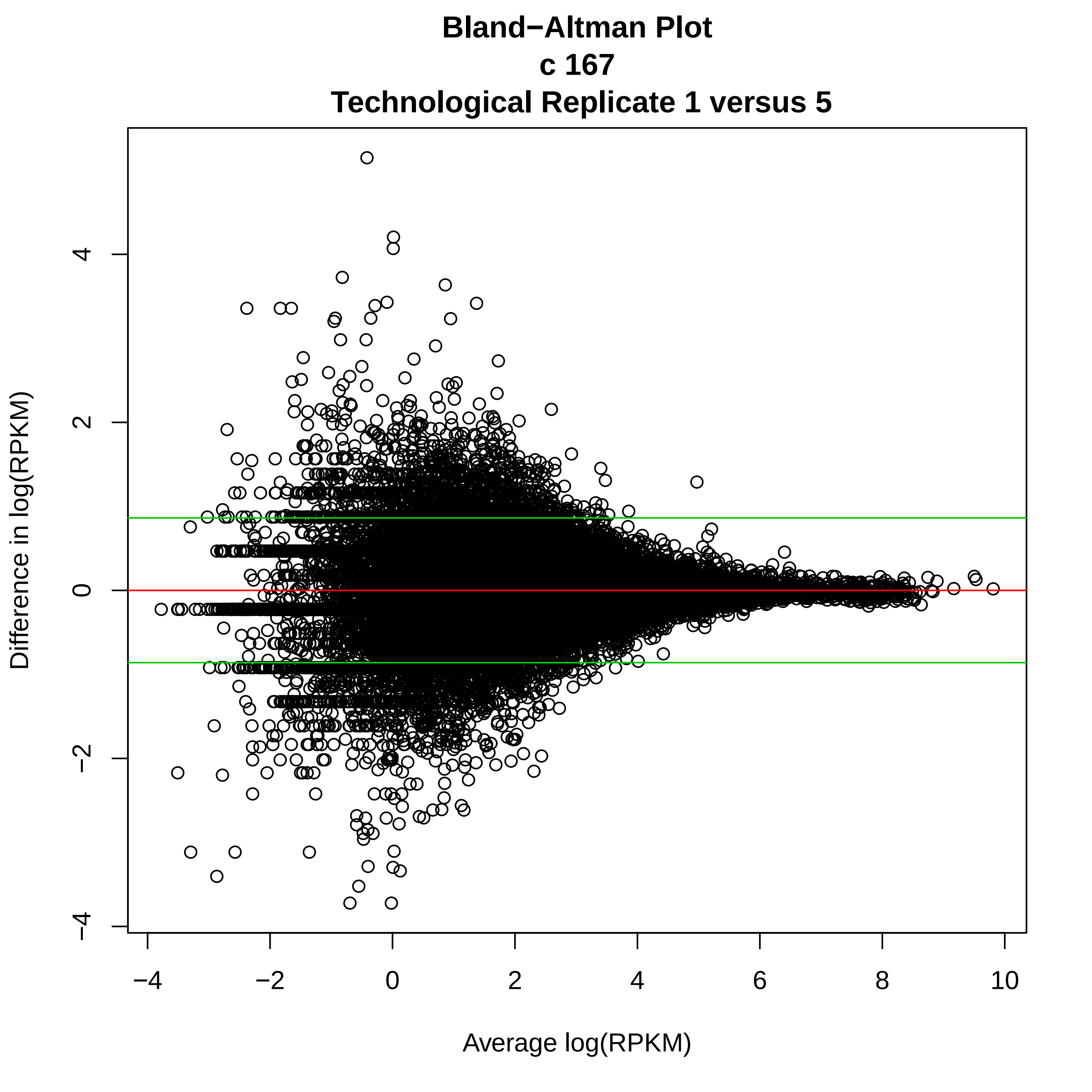

Bland−Altman Plot
c 167
Technological Replicate 1 versus 5
Difference in log(RPKM)
Average log(RPKM)
−4
−2
0
2
4
6
8
10

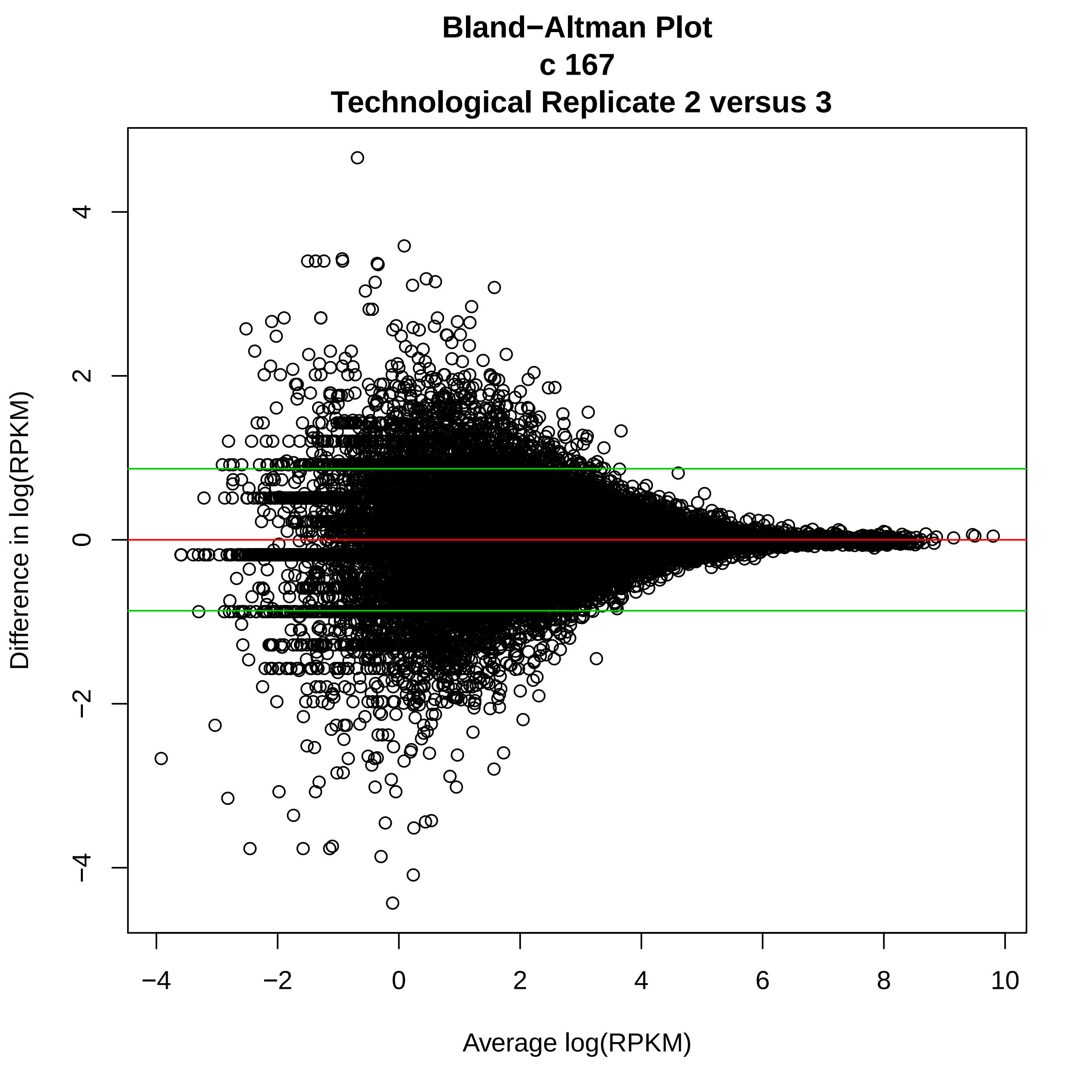
Bland−Altman Plot
c 167
Technological Replicate 2 versus 3
Difference in log(RPKM)
Average log(RPKM)
−4
−2
0
2
4
6
8
10

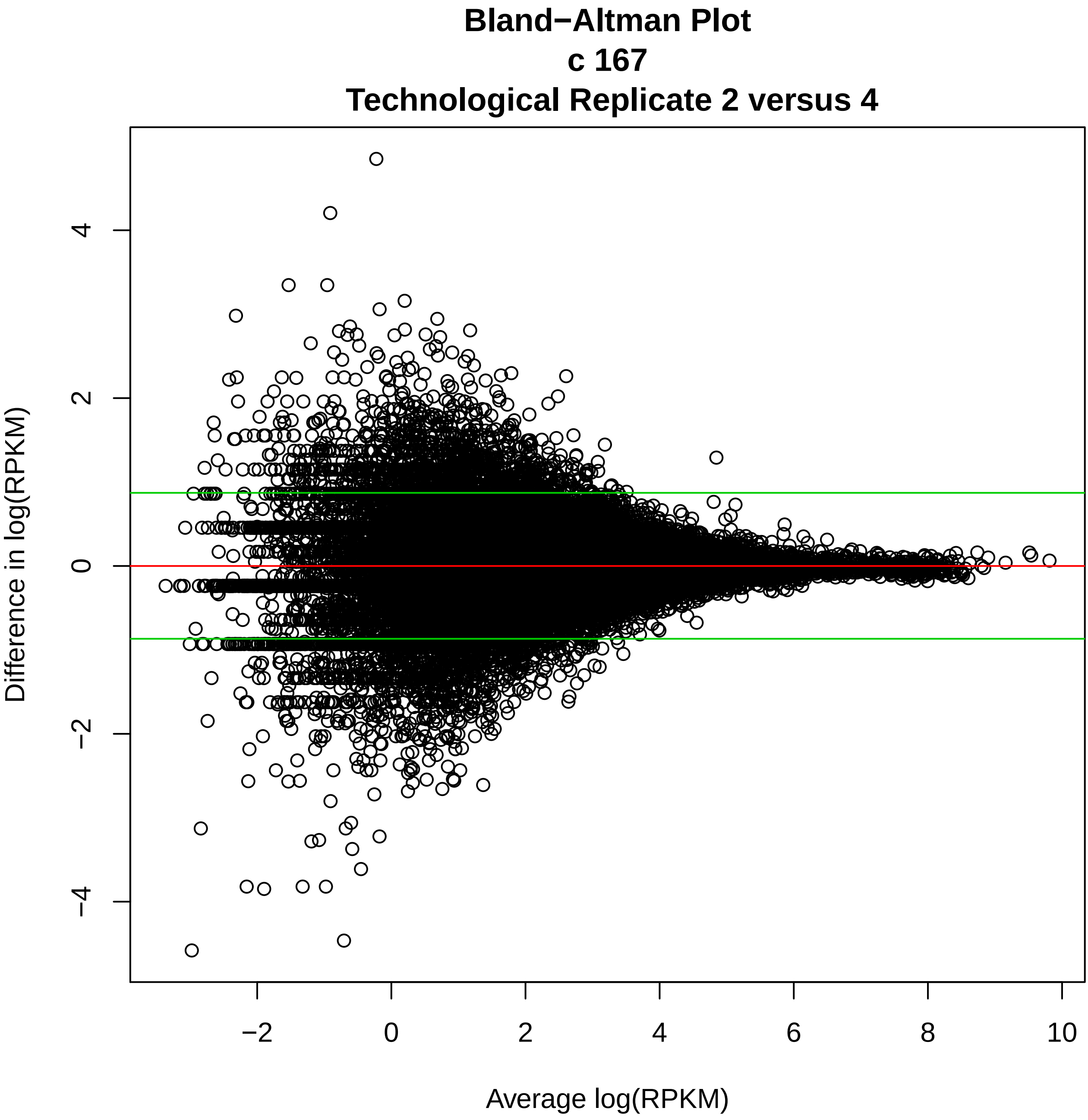

Bland−Altman Plot
c 167
Technological Replicate 2 versus 4
Difference in log(RPKM)
Average log(RPKM)
−4
−2
0
2
4
−2
0
2
4
6
8
10

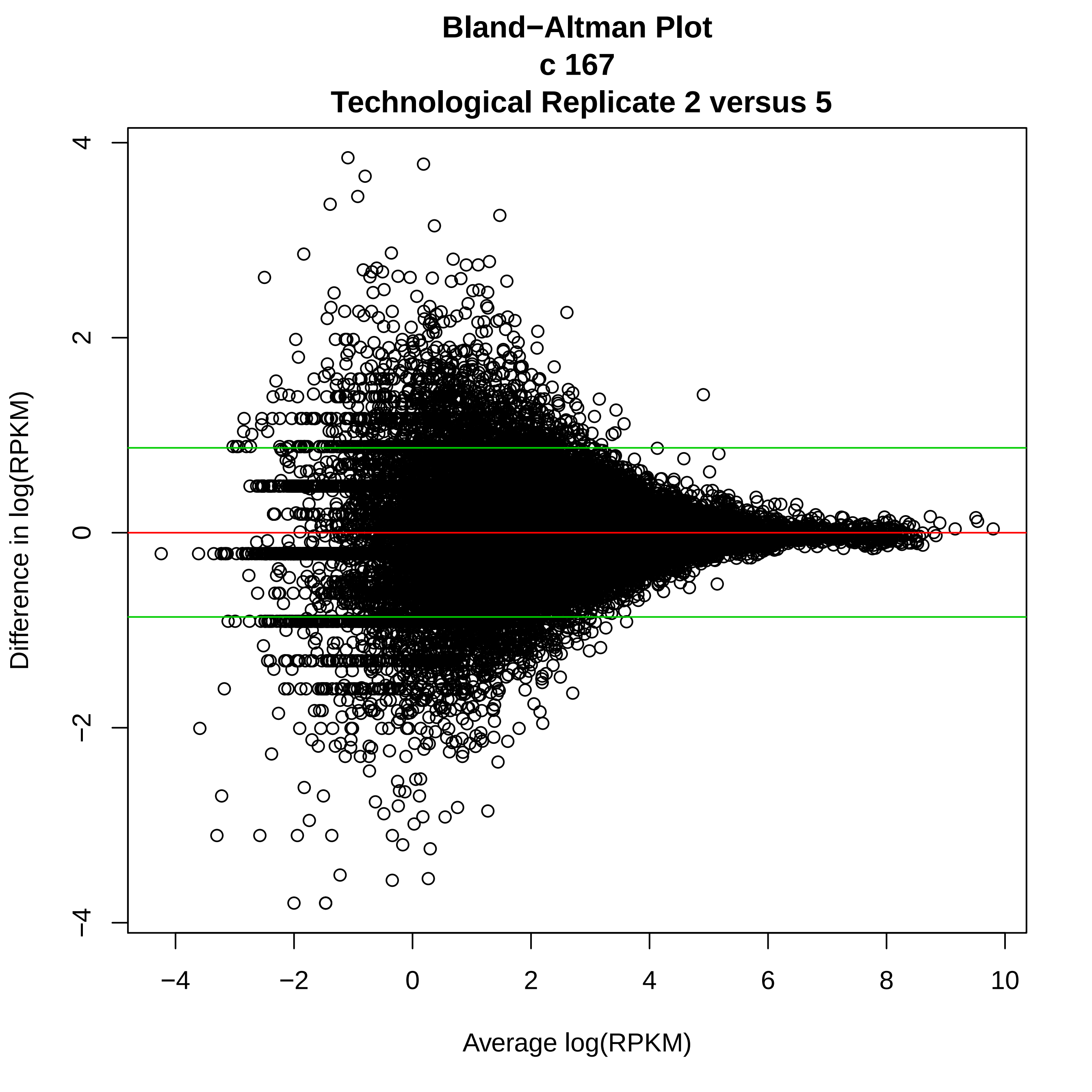

Bland−Altman Plot
c 167
Technological Replicate 2 versus 5
Difference in log(RPKM)
Average log(RPKM)
−4
−2
0
2
4
6
8
10

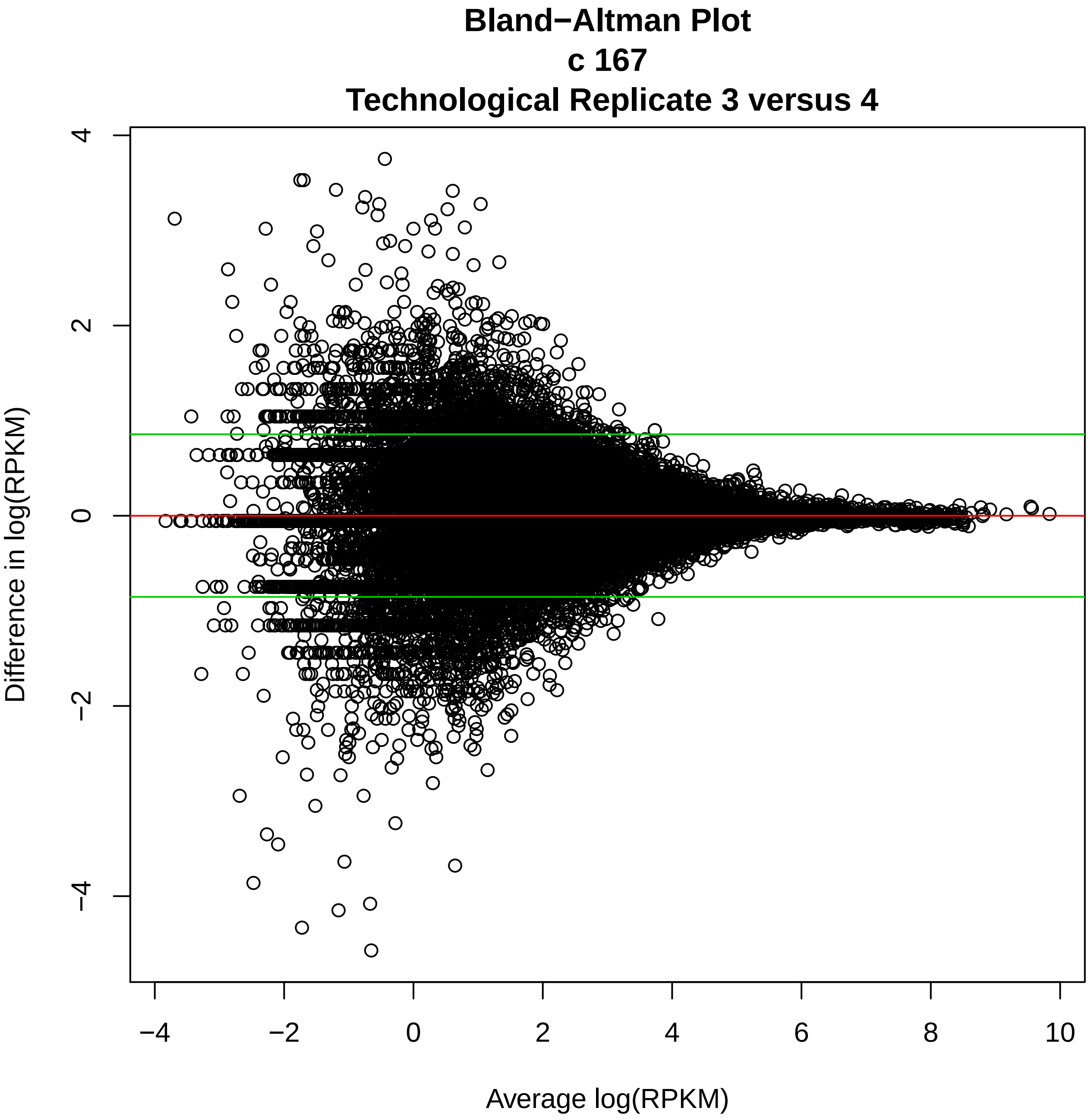

Bland−Altman Plot
c 167
Technological Replicate 3 versus 4
Difference in log(RPKM)
Average log(RPKM)
4
2
0
−2
−4
−4
−2
0
2
4
6
8
10

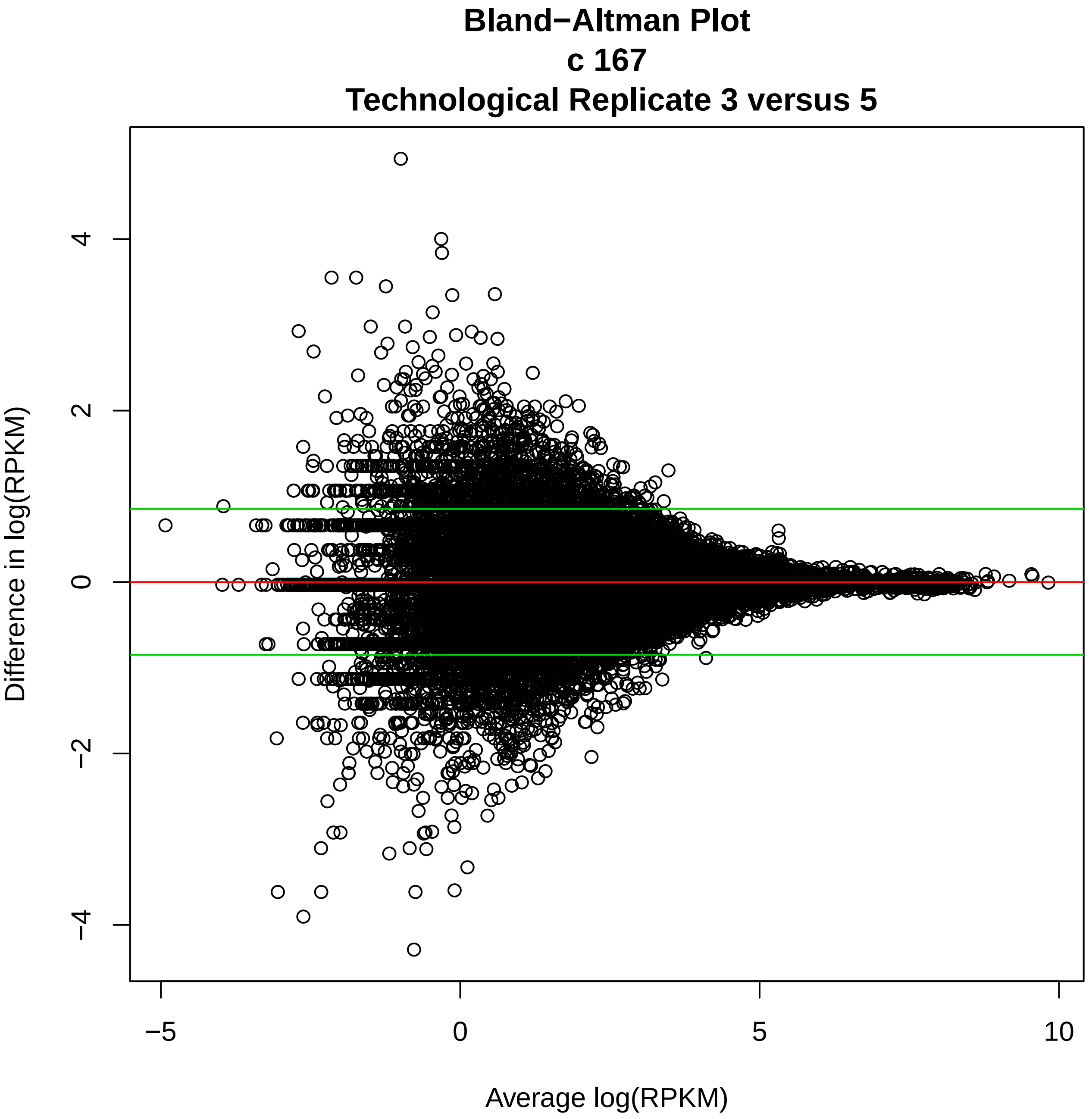
Bland−Altman Plot
c 167
Technological Replicate 3 versus 5
Difference in log(RPKM)
Average log(RPKM)
4
2
0
−2
−4
−5
0
5
10

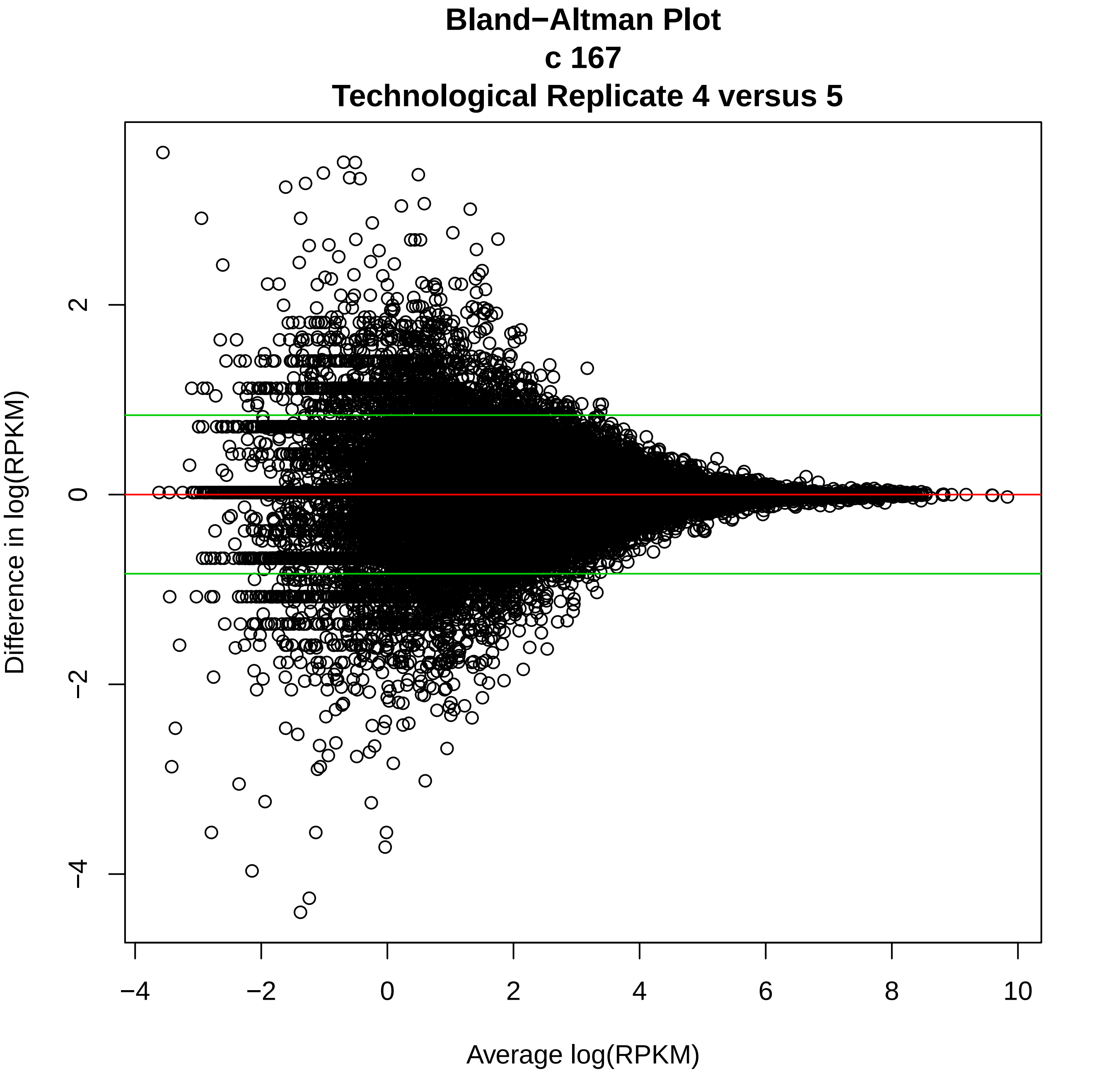
Bland−Altman Plot
c 167
Technological Replicate 4 versus 5
Difference in log(RPKM)
Average log(RPKM)
−4
−2
0
2
4
6
8
10

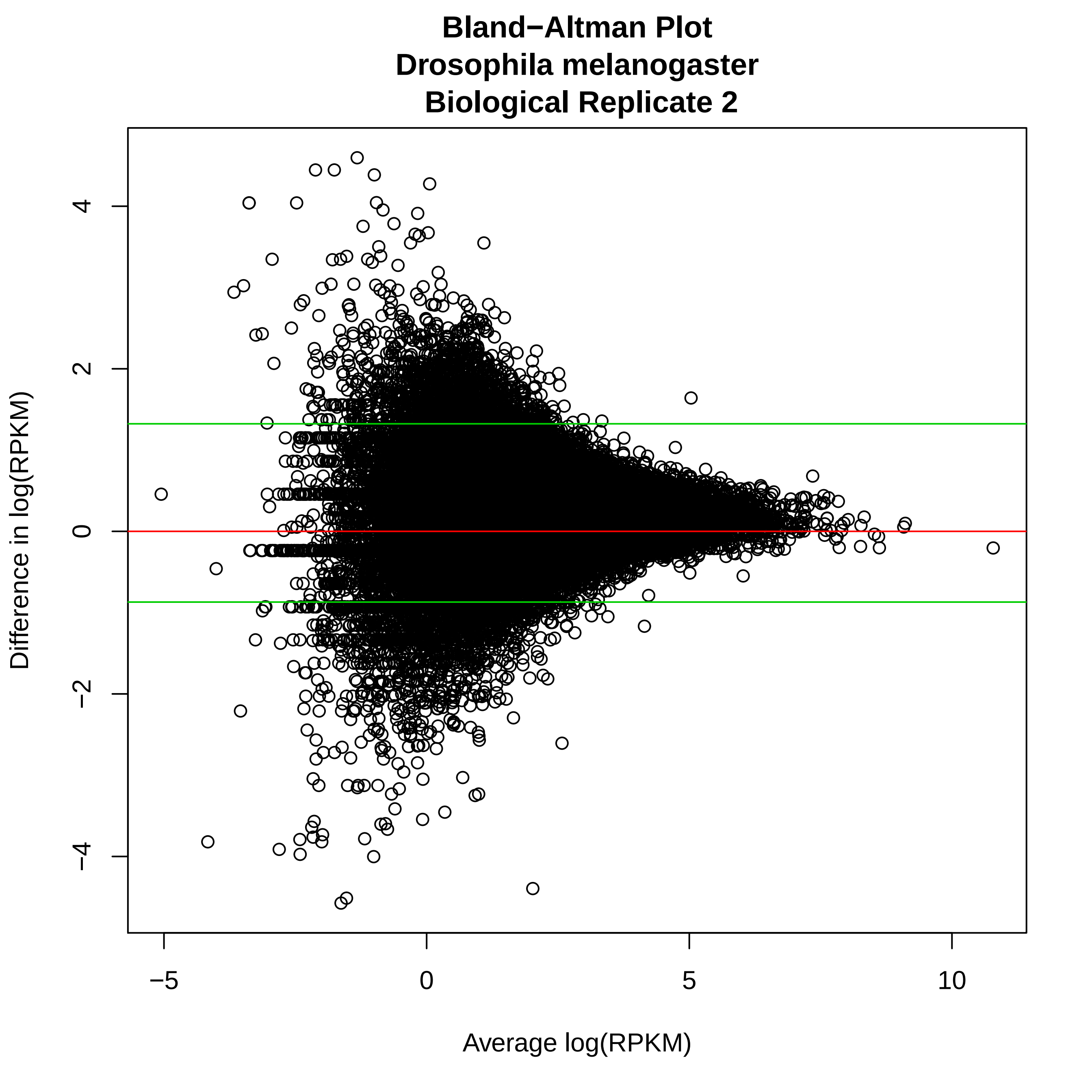
Bland−Altman Plot
Drosophila melanogaster
Biological Replicate 2
Difference in log(RPKM)
Average log(RPKM)
−4
−2
0
2
4
−5
0
5
10

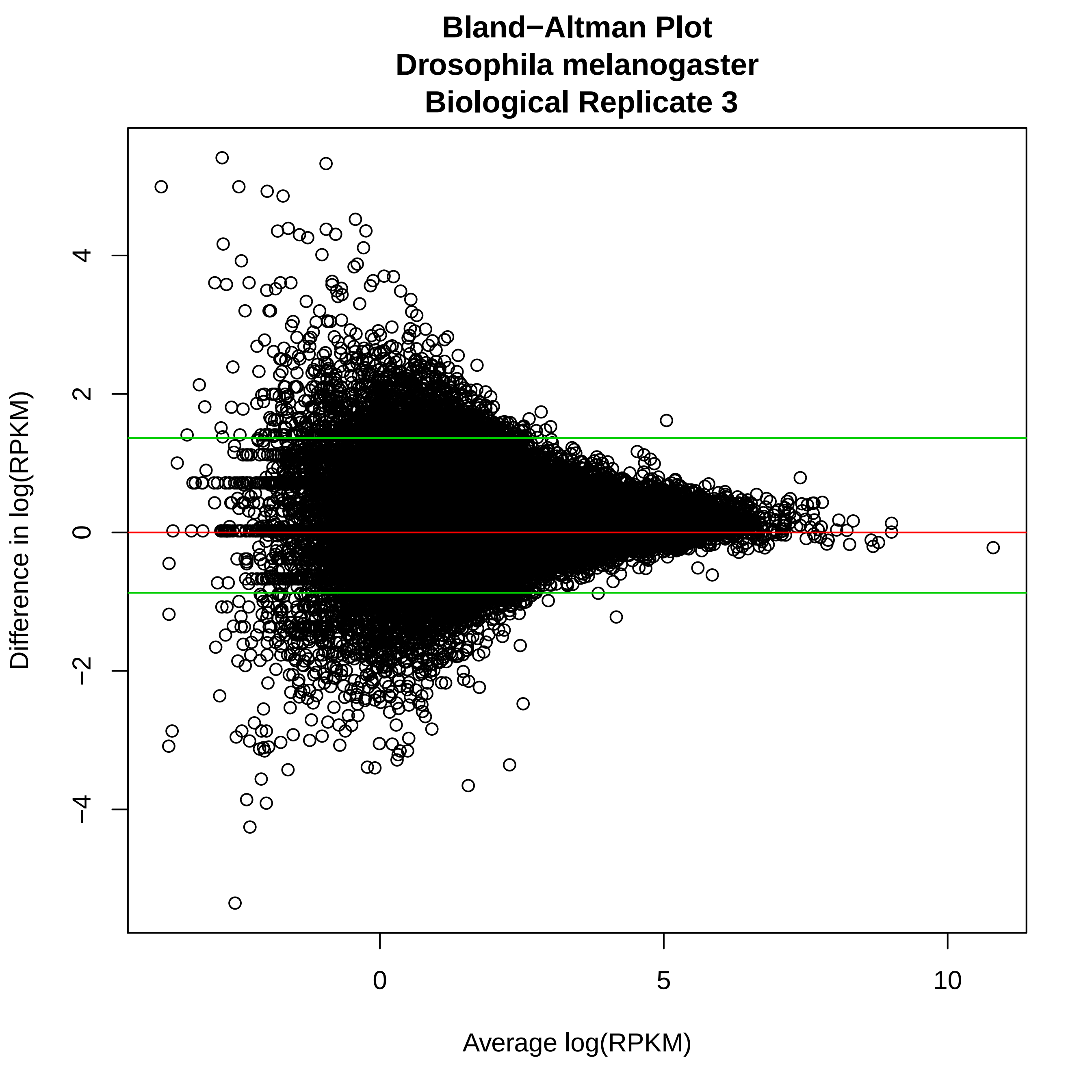

Bland−Altman Plot
Drosophila melanogaster
Biological Replicate 3
Difference in log(RPKM)
Average log(RPKM)
4
2
0
−2
−4
0
5
10

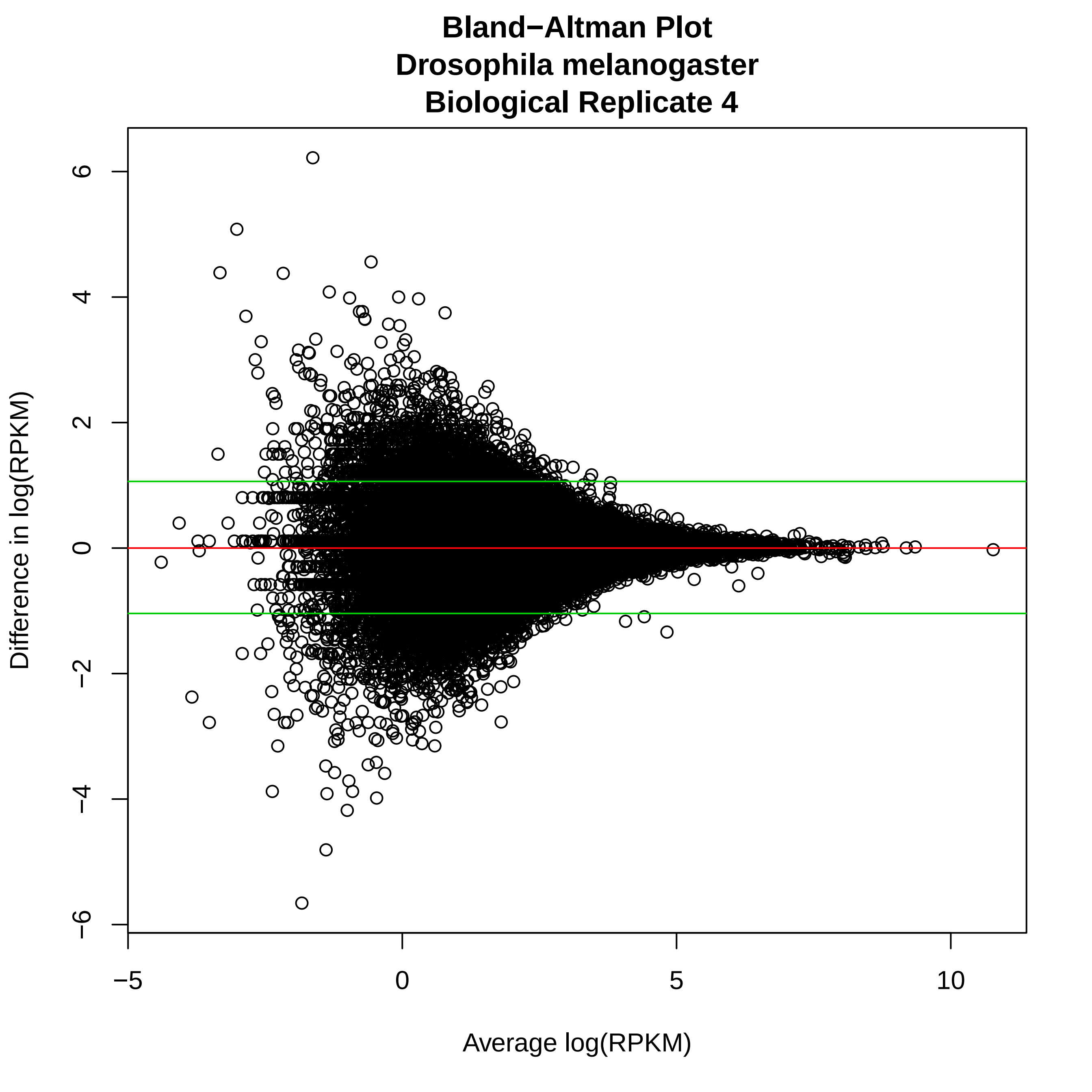
Bland−Altman Plot
Drosophila melanogaster
Biological Replicate 4
Difference in log(RPKM)
Average log(RPKM)
−6
−4
−2
0
2
4
6
−5
0
5
10
